# Hexane extract of *Plumbago europaea* L. aerial parts: phytochemical screening and antibacterial activity

**DOI:** 10.1039/d5ra07370g

**Published:** 2026-04-10

**Authors:** Muhannad Hasan, Nidal Hasan, Nathalie Moussa, Imad Hwija, Yaseer Mossa, Abdel Nasser Singab

**Affiliations:** a Faculty of Dentistry, Manara University Lattakia Syria mohannad.hasan@manara.edu.sy; b Department of Life Sciences, Faculty of Sciences, Tishreen University Lattakia Syria; c Department of Pharmaceutical Chemistry and Drug Control, Faculty of Pharmacy, Manara University Lattakia Syria; d Department of Chemistry, Faculty of Sciences, Tishreen University Lattakia Syria; e Department of Pharmacognosy, Ain-Shams University Cairo Egypt; f Centre of Drug Discovery Research and Development, Ain Shams University Cairo Egypt

## Abstract

*Plumbago europaea* L. is a plant that is commonly used in Syrian folk medicine. This study aimed to identify the chemical constituents of the hexane extract from the plant's aerial parts and evaluate its antibacterial efficacy for the first time. The aerial parts (flowers and leaves) of the plant were collected from a mountainous area in Lattakia Governorate, Syria. This was followed by extraction using the Soxhlet apparatus with hexane solvent. Then the chemical composition of this extract was determined using a Headspace Gas Chromatography–Mass Spectrometry apparatus (HS/GC-MS). Moreover, the antibacterial activity of the hexane extract was studied using the disc diffusion method against two types of bacteria: *Escherichia coli* (Gram-negative) and *Staphylococcus aureus* (Gram-positive). 63 and 52 compounds were identified in the hexane extract of both flowers and leaves, respectively. The main compounds in the flower extract were campesterol (7.31%), heneicosanol (6.81%), and phytyl decanoate (6.13%) β-amyrone (5.77%), β-amyrin (5.67%), plumbagin (5.67%). While the main compounds in the leave extract were campesterol (10.26%), heneicosanol (9.00%), β-amyrone (8.33%), plumbagin (7.75%). In addition, the disc diffusion test findings also demonstrated that the hexane extract of *P. europaea* L. aerial parts was clearly efficient against the tested bacteria at varying doses. Additionally, the flower extract had a stronger effect on the studied bacteria than the leaves, and it beat different antibiotics. The intriguing search results lay the foundation for further chemical and biological research that will help identify beneficial new uses for this plant in medicine and nutrition.

## Abstract 2

*Plumbago europaea* L. is a plant that is commonly used in Syrian folk medicine.

## Graphical abstract

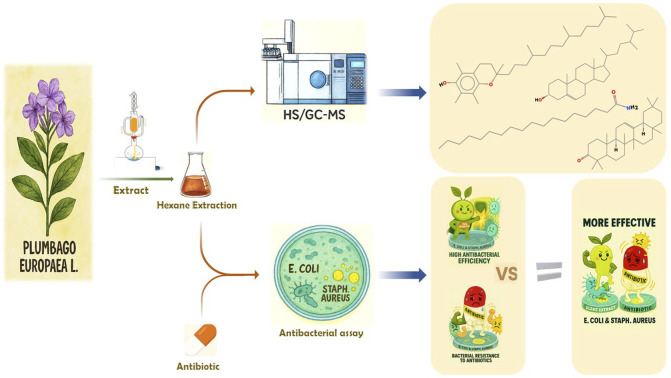

## 1. Introduction

Infectious diseases are the world's greatest cause of death, accounting for an estimated 50 000 fatalities annually. This is exacerbated by the arrival of novel drug-resistant microbial strains. Bacteria are the primary cause of most infectious disorders.^1^ Numerous bacteria have demonstrated unique drug resistance, possessing the capacity to quickly evolve a resistance mechanism through spontaneous mutations and transmit these traits *via* horizontal gene transfer. As a result of the bacteria's quick evolution, antibiotic resistance rises. Furthermore, multidrug-resistant bacteria have proliferated as a result of antibiotic abuse and the inability to develop new antibiotics. Growing public health concerns have resulted from this.^2–4^ Therefore, in order to protect humanity from the threat of multidrug-resistant infectious diseases, researchers worldwide must work tirelessly to find new medications, antibiotics, or preparations from a variety of sources that can be used to effectively control the spread of multidrug-resistance.^5^

Humans have historically utilized plants as a source for drug development or as a botanical remedy to cure a variety of illnesses.^6^ However, most pharmaceutical companies have turned their primary attention towards purified synthetic or semi-synthetic chemical compounds in recent decades due to the basic challenges connected with the isolation of medications derived from natural materials. Sadly, the outcomes fell short of goals for world health.^7^ This has contributed to increasing awareness of the use of medicinal plants in recent years because of their potential to treat a wide range of recognized and emergent illnesses.^8^ It has been discovered that several medicinal plants' crude extracts have strong antibacterial qualities against a wide variety of microbes.^9^ Plant extracts have an effect on a variety of bacteria because they contain a vast number of chemical components that are biologically active and combine to harm microorganisms.^10^ Thus, interests around the world have once again turned to the creation of medications derived from natural sources. Starting pharmacological research with raw plant extracts and subsequently isolating and characterizing the constituents that give the extract its pharmacological activity has become standard practice.^11^

The *Plumbaginaceae* family is notable for its nutritional and therapeutic value since it includes flavonoids, terpenoids, and naphthoquinone, all of which have different biological activities. In the hunt for novel medications, extracts from some species in this family and chemicals that have been separated from them have proven to be quite significant.^12^ One of the species in this family is *P. europea* L., also referred to locally as the AL-assab plant. It is a perennial herbaceous plant with an upright stem that is between 30 and 100 cm long. The plant has green leaves, with the lower leaves being elliptical or serrated and the upper leaves being sessile. Its purple flowers bloom from May to August. The nations of the northern and western Mediterranean region are its native home. It grows at elevations between 650 and 2200 meters on rocky soils, in limestone, on roads, slopes, dry hills, and mountainsides.^13^

*P. europea* L. is used. In folk medicine as an antidote to cancer, rheumatism, dysmenorrhea, and inflammation, and to treat respiratory, dental, skin, and other diseases.^14–21^ Although the plant is widely used in folk medicine, there hasn't been much research showing how well its extracts work against illnesses and infections. The methanolic extract of the plant's leaves showed *in vitro* antioxidant and antiobesogenic activity,^21^ whereas a laboratory investigation employing aqueous plant extracts showed a diuretic effect.^7^ According to the findings of another study on the plant's methanolic extract's impact on mouse stem cells, the extract itself induces mutations, but at low concentrations it is probably antimutagenic and can change genetic mutations in mice.^22^ A plumbagin molecule that was isolated from the roots of plants of this species has also been found to have cytotoxic, antibacterial, and antifungal activities,^23^ as well as antimalarial, antimicrobial, anticancer, and antifertility characteristics.^24^ Furthermore, the ethanolic extract and plumbagin chemical that were isolated from the plant stem completely inhibited the dermatophytes that were examined, indicating that this plant can be used to fight dermatophytes.^25^

Phytochemical investigations on *P. europaea* L. remain notably scarce. Only a single study has reported a phytochemical screening of polar organic extracts (acetone and methanol) from the plant's roots, revealing the presence of cardiac glycosides, alkaloids, phenolic compounds, tannins, and sterols.^21^ In contrast, phytochemical screening of various organic extracts from other *Plumbago* genus has demonstrated substantial variability in chemical classes, influenced by plant species, plant part, and the solvent employed. Polar organic extracts from different organs of *P. zeylanica* L. revealed glycosides, flavonoids, saponins, tannins, alkaloids, terpenes, and phenolics in the methanolic extract of leaves and stems. Meanwhile, the ethanolic extract of the roots contained sterols alongside the aforementioned constituents.^26–28^ Furthermore, the methanolic root extract of *P. indica* was distinguished by the presence of all previously identified chemical classes except terpenes.^29^ On the other hand, the methanolic stem extract of *P. auriculata* was limited to flavonoids, alkaloids, and phenolics.^30^ As for the non-polar organic extracts (ethyl acetate, chloroform, petroleum ether, and diethyl ether) of the leaves, roots, and stems of *P. zeylanica* L., all were found to contain sterols, terpenes, and glycosides, while the presence of other chemical classes varied among the extracts.^26–28^ Additionally, a single study was conducted to determine the chemical composition of the ethanolic extract from the leaves, stems, and roots of *P. europaea* L. using GC/MS analysis. The results identified plumbagin as the major compound, along with terpenes and sterols as principal constituents.^25^ Moreover, similar findings were reported in studies that investigated the chemical composition of organic extracts (ethyl acetate and methanol) from other *Plumbago* genus, including *P. indica*,^29,31^*P. zeylanica* L.,^32^*P. auriculata*, and *P. scandens*,^30^ also using GC/MS techniques.

To our knowledge, no prior research has been done on the chemical composition of plant extracts of aerial parts from *P. europea* L. As we previously indicated, there aren't many studies on the biological efficacy of its organic extracts, and those that exist are restricted to polar extracts. Therefore, due to this plant's medicinal value and as a follow-up to our previous studies on the essential oils of its aerial parts,^33^ this study aimed to perform a phytochemical screening and investigate the biological activity against bacteria on the hexane extract of the aerial parts of the Syrian *P. europea* L. for the first time.

## 2. Experimental

### 2.1 Instrumentation and chemicals

#### 2.1.1 Apparatus

HS/GC-MS (Shimadzu QP2020, Japan), GC-FID (Shimadzu GC-2010, Japan), Lyophilizer (CHRIST, ALPHA 1–2, Germany), Soxhlet (ISOlab, Germany), Autoclave (Memmert, A160, Germany), Incubator (Memmert, IN30, Germany), Rotary evaporator (Heidolph, Laborota 4010, Germany), Drying oven (Jant, Locally Manufactured), analytical balance *d* = 0.01 g (Sartorius, Practum3102-1S, Germany), laboratory flat electric hot plate (Gerhardt, HC63, Germany), laboratory heating mantle (Electromantle, EM1000/CEX1, Germany), UV lamp (CAMAG AG, UV Lamp 4, Switzerland), Freezer (Kelvinator, KLAF675B-E20B, China), microbial culture room (Locally Manufactured), micropipette (Dragonlab, DV40223, China), laboratory glassware (Isolab, Germany), plastic and glass Petri dishes (9 cm diameter), metal forceps, alcohol bunsen burners, microbial culture tubes, metal inoculating loop.

#### 2.1.2 Chemicals

Sulfuric acid, acetic acid, glacial acetic acid, hydrochloric acid, lead acetate, mercuric chloride, iron(iii) chloride, potassium iodide, sodium hydroxide, potassium hydroxide, α-naphthol (Sigma-Aldrich, Germany), filter paper (Whatman No. 1, USA).

#### 2.1.3 Bioactive chemicals

Mueller Hinton Agar, EMB Agar, Nutrient broth (Merck, Germany).

#### 2.1.4 Antibiotics

Tobramycin, levofloxacin, clindamycin, iImipenem, ampicillin, doxycycline, gentamicin, cefalexin, cefepime, tetracycline, amikacin, azithromycin, vancomycin, trimethoprim/sulfamethoxazole, ciprofloxacin, amoxycillin (Microexpress, Biogram, India).

### 2.2 Plant materials

The aerial parts (flowers and leaves) of the wild plant *P. europaea* L. were collected during the flowering period in June 2024 from a mountainous region in Lattakia Province, Syria (35°29′19″N, 36°10′36″E; approximately 900 m altitude). A voucher specimen (No. HFS 231) has been deposited in the herbarium of the faculty of science (Tishreen University, Lattakia, Syria). Plant specimens were identified by Prof. Dina Haddad from that faculty. After washing and drying in the shade for 25 days, they were ground well and stored in an opaque glass bowl up until extraction.

### 2.3 Extraction

The aerial parts (flowers and leaves) of *P. europaea* L. were extracted following the methods outlined,^34^ with a few modifications utilizing a Soxhlet apparatus and a hexane solvent. A total of 400 g of plant material was extracted with 1000 mL of hexane over 10 hours, and the extraction was repeated three times. Following filtration with Whatman No. 1 filter paper, the extracts were vacuum-concentrated in a Rotary evaporator set to 30 °C and 140 rpm. Then, the extracts were dried with a Lyophilizer apparatus. The dry extracts weighed 16.91 ± 1.9 g for flowers and 18.80 ± 2.3 g for leaves. The extract yield (w/w%) was calculated by dividing the dry extract weight by the initial dry plant powder weight. The resulting dry extracts were then kept at +4 °C in dark glass containers until chemical and biological activity analyses.

### 2.4 Preliminary phytochemical screening

The hexane extract of the aerial parts of *P. europaea* L. was screened for the presence of secondary metabolites, including terpenoids, steroids, saponins, tannins, alkaloids, flavonoids, glycosides (including cardiac glycosides), and coumarins. The screening procedures were conducted with slight modifications to the standard protocols described by Trease and Evans,^35^ and Harborne.^36^

### 2.5 HS/GC-MS analysis of organic extracts

The hexane extracts were subjected to HS/GC-MS analysis in accordance with previously established standard protocols,^37^ with minor modifications. The HS/GC-MS analysis was conducted at the Drug Discovery Research and Development Center, Faculty of Pharmacy, Ain Shams University, Cairo, Egypt, using a Shimadzu GCMS-QP2020 system, comprising a GC-2010 unit equipped with an AOC-20i autosampler, coupled to a QP2020 mass spectrometer. The system was fitted with an RTX-1MS capillary column (30 m × 0.25 mm i.d. × 0.25 µm film thickness). The analysis was conducted under electron impact (EI) mode with an ionization energy of 70 eV. Helium was employed as the carrier gas at a flow rate of 1.37 mL min^−1^, with a purge flow of 3.0 mL min^−1^, a pressure of 80.0 kPa, an equilibrium time of 3.0 min, and a linear velocity of 42.5 cm s^−1^. Sample injection was performed in split mode at a ratio of 30 : 1. The injector temperature was maintained at 280 °C, while the ion source temperature was set at 220 °C. The oven temperature was programmed from 50 °C (held for 3.0 min) to 300 °C (held for 10.0 min) at a ramp rate of 5 °C min^−1^, with a solvent cut time of 2.0 min. Samples were dissolved in hexane, and 1 µL of each sample was injected. Mass spectra were acquired at 70 V over a scan range of 35.00–500 *m*/*z*, with a scan interval of 0.30 s per scan. The total HS/GC-MS run time was 63.00 min. The samples were injected in triplicate (*n* = 3).

The compound identification was performed by comparing the experimental mass spectra with the NIST17 s.lib and Adams^38^ databases and calculating the Linear Retention Index (LRI) according to the Van den Dool and Kratz^39^ method, using a mixture of *n*-alkanes (C8–C35), which were injected under identical chromatographic conditions. Components were considered tentatively identified when both spectral match (similarity ≥85%) and LRI deviation (≤±10 units) criteria were met. Although confirmation with authentic standards would further increase the confidence in compound identification, the determination of retention indices and their comparison with available reference databases, together with mass spectral data, represents the standard and widely accepted approach for compound identification in the phytochemical analysis.^40,41^

### 2.6 Gas chromatography/flame ionization detector (GC-FID)

GC-FID was used to quantify the hexane extract of *P. europaea* L., using the same chromatographic conditions and column described above. The relative percentage of each compound was calculated from the area of the corresponding peak in the chromatogram, normalized to the total area of all the detected peaks.

### 2.7 Antibacterial assays

#### 2.7.1 Bacterial strains

Initially, clinical isolates of *E. coli* (from a urine sample) and *S. aureus* (from a wound swab) were obtained from the microbiology laboratory at Tishreen University Hospital. These isolates represent Gram-negative and Gram-positive bacteria, respectively. Subsequently, the bacteria were cultivated and purified using various culture media. To identify the strains, biochemical tests were conducted, followed by classification using the API 20E system and Bergey's Manual of Systematic Bacteriology.^42^ The cultures were maintained on Nutrient agar in screw-cap bottles and stored at 4 °C. To ensure viability and purity, all cultures were periodically subcultured. Test cultures were prepared by transferring a loopful of bacteria from the original nutrient broth cultures onto Petri dishes containing Mueller–Hinton Agar (MHA). The plates were then incubated at 37 °C for 24 hours.

#### 2.7.2 Antibiotic susceptibility testing

The susceptibility of the isolated bacterial strains to 16 different antibiotics (as listed in Table 1) was evaluated using the disc diffusion method, commonly known as the Bauer–Kirby technique (1966).^43^ This method relies on the diffusion of the antibiotic from a paper disc into MHA, the standard culture medium. A bacterial suspension was prepared for each isolate at a turbidity of 0.5 McFarland standard, equivalent to approximately 1.5 × 10^8^ cells per mL. Then, 0.1 mL of the suspension was evenly spread onto MHA plates using a sterile cotton swab. Antibiotic discs were subsequently placed on the surface of the agar, and the plates were incubated at 37 °C for 24 hours. The antimicrobial susceptibility of the pathogens was assessed by measuring the diameters of the inhibition zones around each disc and comparing them to the interpretive standards provided in the reference tables for each antibiotic. These antibiotics were chosen for the study due to their widespread use and ability to function in accordance with the four known mechanisms of antibiotic activity.^44^

**Table 1:** Antibiotics used to test the investigated bacterial isolates for susceptibility

No.	Antibiotics	Code	Conc. mg per disk
1	Cefepime	CPM30	30
2	Vancomycin	VA30	30
3	Amoxycillin	AMC20	20
4	Trimethoprim/Sulfamethoxazole	COT25	25
5	Gentamicin	GEN10	10
6	Cefalexin	CN30	30
7	Levofloxacin	LE5	5
8	Imipenem	IPM10	10
9	Ampicillin	AMP10	10
10	Tobramycin	TOB10	10
11	Clindamycin	CD2	2
12	Doxycycline	DOX30	30
13	Amikacin	AK30	30
14	Ciprofloxacin	CIP5	5
15	Azithromycin	AZM15	15
16	Tetracycline	TE30	30

#### 2.7.3 Antibacterial bioassay procedure

The inhibitory activity of the hexane extract of the *P. europaea* L. aerial parts was evaluated using the agar disc diffusion method (Kirby–Bauer technique). A stock solution was prepared by dissolving 1000 mg of the dried extract in 1 mL of hexane. Serial dilutions were then performed to obtain the desired concentrations. Sterile filter paper discs (Whatman No. 1, 6 mm diameter, 1 mm thickness) were impregnated with 20 µL of each prepared concentration. The concentrations and their corresponding disc loads are presented in Table 2. Additional sterile discs were impregnated with 20 µL of pure hexane to serve as negative controls. All discs were left at room temperature to allow solvent evaporation. A single colony from each 24 hour-old bacterial culture grown on solid medium was used to prepare a bacterial suspension in physiological saline, adjusted to a turbidity of 0.5 McFarland standard (≈1.5 × 10^8^ cfu mL^−1^). Then, 0.1 mL of the suspension was evenly spread onto MHA plates (prepared according to standard protocols) using a sterile cotton swab. The loaded discs were placed on the agar surface and refrigerated for 2 hours to allow diffusion of the active compounds. Plates were then incubated at 37 °C for 24 hours. The presence of a clear inhibition zone around the discs indicated antibacterial activity. Inhibition zone diameters were measured using a millimeter ruler (Piacolys). The bacterial response to the tested extracts was classified based on inhibition zone diameters as follows: resistant (R): no zone, weak sensitivity: 7–10 mm, moderate sensitivity: 10.1–15 mm, high sensitivity: 15.1–30 mm.^45–47^ All experiments were performed in triplicate.

**Table 2:** Hexane extract concentrations of *P. europaea* L. aerial parts applied in the antibacterial activity evaluation^a^

No.	Conc. (mg mL^−1^)	Loaded extract per disc (mg)
1	1000	20
2	500	10
3	250	5
4	125	2.5
5	62.5	1.25
6	32.125	0.625
7	15.62	0.312
8	7.81	0.156

a Each disc was loaded with 20 µL of the prepared plant extract solution.

### 2.8 Statistical analysis

All bioassays were conducted in triplicate, and the data were reported as means ± standard deviation (SD) (*n* = 3). A two-way analysis of variance (two-way ANOVA) was performed to evaluate the pairwise interactions between variations in antibiotics and bacterial strains and their effects on the mean diameters of inhibition zones. A three-way analysis of variance (three-way ANOVA) was also conducted to assess both pairwise and three-way interactions among variations in plant part, concentration, and bacterial strain, and their influence on the mean inhibition zone diameters using univariate analysis. Furthermore, a one-way analysis of variance (one-way ANOVA) was performed to evaluate the effect of varying concentrations of each plant part against each bacterial strain, as well as to assess the impact of different concentrations of the flower extract and the tested antibiotics on the bacterial strains. Resistant outcomes—defined as the absence of an inhibition zone—were assigned a value of 0 mm for statistical processing. To ensure that recoding resistant outcomes as 0 mm did not introduce artificial variance compression, ANOVA models were examined before and after the transformation. The error variance, effect size estimates, and significance patterns remained consistent across analyses. These diagnostics indicate that the transformation did not alter the variance structure or affect the validity of the statistical inferences. Following this transformation, ANOVA assumptions were evaluated, homogeneity of variances was assessed using Levene's test, and statistical power was estimated using G*Power version 3.1.9.4. Tukey's honestly significant difference (HSD) tests were subsequently applied for pairwise comparisons and identification of homogeneous groups. An independent-samples *t*-test was also performed to compare the concentrations of the flower and leaf extracts against the bacterial strains. Differences were considered statistically significant at *p* < 0.05. All statistical analyses were performed using IBM SPSS Statistics version 27.

## 3. Results and discussion

### 3.1 Plant extract yield

The Soxhlet extraction of *P. europaea* L. aerial parts with hexane as a solvent produced semi-solid organic extracts with a strong herbal scent and a dark brown hue. The extraction yields were 4.22% for the flowers and 3.69% for the leaves.

### 3.2 Preliminary phytochemical screening

Tests performed on the hexane extract of *P. europaea* L.'s aerial parts revealed that it was high in steroids, terpenoids, and glycosides (particularly cardiac), while coumarins and saponins were present in trace amounts. The leaves extracts contained modest levels of tannins, whereas the flowers extracts did not contain any. Moreover, both alkaloids and flavonoids were absent from the extracts, as shown in the Table 3.

**Table 3:** Preliminary phytochemical screening results of the hexane extract from the aerial parts of *P. europaea* L^a^

Secondary metabolites	Test	Aerial part	
		Flowers	Leaves
Terpenoids		+++	+++
Steroids	Liebermann–Burchard	+++	+++
Saponins	Foam	+	+
	Mercuric chloride	+	+
Tannins	Ferric chloride	−	++
	Lead acetate	−	++
Alkaloids	Mayer's	−	−
	Wagner's	−	−
Flavonoids	Alkaline reagent	−	−
Glycosides	Molisch's	+++	+++
Cardiac glycosides	Keller–Killiani	+++	+++
Coumarins		++	++

a +++: high concentration, ++: medium concentration, +: low concentration, −: absent.

As observed, the hexane extracts of the flowers and leaves of *P. europaea* L. exhibited similar profiles in terms of their chemical constituents. When compared with previous studies conducted on various non-polar organic extracts from other *Plumbago* genus,^26–28^ the presence of sterols, terpenes, and glycosides was noted as major constituents. However, the occurrence of other chemical classes varied among the extracts. This similarity may be attributed to the fact that these plants belong to the same genus; this may reflect shared biosynthetic pathways among species of the same genus, in addition to the comparable nature of the solvents used. Conversely, differences in plant species, plant part, and the type of solvent employed may contribute to the observed variation in the presence of other chemical classes.^48,49^

### 3.3 HS/GC-MS analyses

A total of 56 and 49 compounds were identified in the hexane extracts of the flowers and leaves, respectively, of *P. europaea* L., using the HS/GC-MS technique. These compounds accounted for 92.22% and 92.67% of the total peak areas shown in Fig. S1 and S2. The identified constituents are listed in Table S1 according to their elution order from the column.

The major compounds identified in the flower extract were campesterol (7.31%), heneicosanol (6.81%), phytyl decanoate (6.13%), β-amyrone (5.77%), β-amyrin (5.67%), plumbagin (5.67%), nonadecanol (4.02%), 5,9-dimethyl decan-2-one (3.39%), stigmasta-3,5-diene (3.29%), 8,14-cedrandiol (3.22%), citronellol (2.99%), kolavenol acetate (2.91%), hexadecanamide (2.79%), and tetracosanol (2.62%).

In contrast, the major compounds identified in the leave extract were campesterol (10.26%), heneicosanol (9.00%), β-amyrone (8.33%), plumbagin (7.75%), stigmasta-3,5-diene (4.20%), β-amyrin (3.92%), phytyl decanoate (3.65%), kolavenol acetate (3.27%), docosanol (2.88%), β-sitosterol (2.52%), nonadecanol (2.42%), 5,9-dimethyl decan-2-one (2.20%), (*E*)-9-octadecenoic acid, propyl ester (2.20%), and eicosanal (2.07%).

Plant lipid compounds play a fundamental role in plant growth and development.^50^ Given that non-polar hexane was used as the extraction solvent in our study, the resulting plant extracts are expected to consist predominantly of lipophilic compounds, whose content and composition vary depending on the plant's biotic, abiotic, and genetic conditions.^51^ This is in line with the results of the HS/GC-MS analysis of the hexane extract of *P. europaea* L.'s aerial parts, which showed that lipid compounds of various kinds dominated the extracts' chemical composition at a rate of 77.45% and 71.6% in flowers and leaves, respectively. The lipid content was characterized by the presence of steroid compounds, accounting for 13.65% and 17.77%, and was primarily compounds of campesterol (7.31%, 10.62%), stigmasta-3,5-diene (3.29%, 4.20%), and β-sitosterol (1.94%, 2.52%) in flowers and leaves, respectively. In addition, fatty alcohols were detected at concentrations of 15.07% and 14.62%, respectively, with the predominant compounds being heneicosanol (6.81%, 9.00%), nonadecanol (4.02%, 2.42%), and docosanol (1.62%, 2.88%) in flowers and leaves, respectively. Furthermore, fatty acids and their esters were present at 5.05% and 5.87%, respectively, with all detected compounds present at concentrations below 2% in both samples. These lipid classes constitute the principal components of cuticular waxes and plant cell membranes. Some of them also function as energy reserves that can be mobilized to support plant growth and development. In addition, they play a protective role against both biotic and abiotic stress conditions.^52–55^ Therefore, their presence is essential for proper plant growth and development, and their elevated concentrations may be attributed either to the plant being in an active growth phase or to its physiological response to environmental stress factors.

On the other hand, the lipid chemical profile of the extracts was distinguished by a high content of terpenoid compounds. Diterpenes were detected at concentrations of 11.8% and 7.47%, represented by the following major constituents: phytyl decanoate (6.13%, 3.65%), and kolavenol acetate (2.91%, 3.27%) in flowers and leaves, respectively. In addition, triterpenes were present at 12.11% and 12.25%, with the predominant compounds being β-amyrone (5.77%, 8.33%) and β-amyrin (5.67%, 3.92%) in flowers and leaves, respectively. In addition, sesquiterpenes were present at 6.51% and 5.48%, with the predominant compounds being 8,14-cedrandiol (3.22%, 1.35%) in flowers and leaves, respectively. Furthermore, monoterpenes were present at 6.38% and 2.93% represented by compounds 5,9-dimethyl decan-2-one (3.39%, 2.2%), and citronellol (2.99%, 0.73%) in flowers and leaves, respectively. These terpenoid compounds are classified as lipophilic substances that do not directly contribute to plant growth and development. However, their elevated concentrations may be attributed to the plant's need for regulation and protection against abiotic stress, as well as their distinctive role in biotic stress responses. Specifically, they serve as key chemical defense agents against pathogens and insect attacks.^56,57^

Fatty acid amides (6.19% for flowers and 3.81% for leaves) were one of the lipid components that set the hexane extract's chemical content apart; these were represented by hexadecanamide (2.79%, 1.52%) in flowers and leaves, respectively. These compounds are known for their defensive role against pathogens and insect herbivory.^58^ Fatty acid amide inducers, produced by various herbivorous caterpillar species, indicate a potential alternative biosynthetic pathway for the occurrence of these compounds. These elicitors stimulate host plants to synthesize and emit volatile organic compounds that serve as chemical signals to attract natural enemies of the herbivores. Consequently, this biosynthetic interaction suggests that fatty acid precursors originate from host plants, whereas caterpillars contribute the amino acid moieties required for amide formation.^59^

Hydrocarbons, on the other hand, were present at 5.28% and 4.38% in flowers and leaves, respectively, with all detected compounds present at concentrations below 2% in both samples.

Moreover, the presence of phenolic compounds (6.81%, 9.41%), represented by plumbagin (5.67%, 7.75%) in flowers and leaves, respectively, and known for their defensive roles against pathogens and insect herbivory,^60^ provides evidence for the plant's chemical composition as a reflection of its exposure to environmental stressors.

A comparison of the chemical composition of hexane extracts from the flowers and leaves of *P. europaea* L. revealed a notable similarity in their major constituents. Such resemblance may be attributed to the fact that both organs belong to the same plant and likely share a single genotype or may modify it in response to the conditions surrounding them. The observed differences in compound concentrations, however, can be attributed to the variability in secretory glands responsible for storing these substances, which vary across plant organs and are influenced by external environmental factors.^61,62^ Furthermore, a comparison was conducted between the chemical composition of hexane extracts from the aerial parts of *P. europaea* L. and the organic extracts from other *Plumbago* genus.^25,29–32^ A similarity was observed in the major chemical classes, particularly the presence of sterols and terpenoids, as previously noted. However, differences were evident in the specific compounds belonging to these classes. In addition, the chemical composition of the hexane extract obtained in the present study differed from that of organic extracts from other *Plumbago* genus in two notable aspects. First, the compound plumbagin was detected at a significantly lower concentration in the hexane extract compared to its reported abundance in organic extracts from previous studies, despite being recognized as a distinctive biochemical marker for this genus. Second, the presence of amide acids in the hexane extract is reported here for the first time in *Plumbago* genus. These differences may suggest a potential alteration in the plant's genetic expression in response to biotic and abiotic stress factors.^63^

Although the primary determinant of a plant's chemical composition is its genetic makeup, which governs biosynthetic processes, the physiological stage at which the plant was collected—namely, the flowering phase—represents a sensitive period during which the plant is exposed to pathogenic agents and pollinators. Moreover, exposure to intense environmental stress and persistent external conditions may induce modifications in the plant's genotype, thereby altering biosynthetic pathways and contributing to both quantitative and qualitative diversity in secondary metabolite production.^63,64^ Accordingly, it can be suggested that the chemical composition of the hexane extracts from the aerial parts of *P. europaea* L. may have undergone genetic modifications in response to insect attack. Supporting this hypothesis, our previous work on the essential oils extracted from the aerial parts of the same plant^33^—collected from the same geographical location and during the same time period—revealed a dominance of phenolic compounds in their chemical composition, which are widely recognized for their defensive role against pathogens and insect herbivory, as previously discussed.

### 3.4 Antibacterial assays

#### 3.4.1 Antibiotic susceptibility of the tested bacterial strains

The isolated pathogenic bacteria were tested for susceptibility to 16 commonly used antibiotics in the treatment of human bacterial infections. The majority of these antibiotics are broad-spectrum and are commonly prescribed for infections caused by both Gram-positive and Gram-negative bacteria, such as skin, respiratory, urinary, and bone infections, among others.^65,66^ As indicated by the results shown in Table 4, *E. coli* exhibited resistance to eight antibiotics, whereas *S. aureus* was resistant to only two and showed good susceptibility to the remaining tested antibiotics. Among the antibiotics against *E. coli*, imipenem demonstrated the highest activity, while tobramycin was the least effective. In contrast, tetracycline showed the greatest efficacy against *S. aureus*, whereas amoxycillin exhibited the lowest.

**Table 4:** Antibiotic susceptibility of the tested *E. coli*^a^

No.	Antibiotic	*E. coli*	No	Antibiotic	*S. aureus*
1	CPM30	0.00^a^	1	CPM30	0.00^a^
2	VA30	0.00^a^	2	AMP10	0.00^a^
3	AMC20	0.00^a^	3	AMC20	11.2 ± 0.6^b^
4	CN30	0.00^a^	4	COT25	14.3 ± 0.5^b, c^
5	AMP10	0.00^a^	5	AK30	17.1 ± 0.8^c, d^
6	CD2	0.00^a^	6	TOB10	17.7 ± 0.8^c, d^
7	CIP5	0.00^a^	7	CN30	18.5 ± 0.9^d,e^
8	TE30	0.00^a^	8	VA30	18.8 ± 0.3^d,e,f^
9	TOB10	10.5 ± 0.2^b^	9	DOX30	20.2 ± 1.2^d,e,f,g^
10	DOX30	11.5 ± 0.3^b^	10	GEN10	21.5 ± 0.7^e,f,g,h^
11	LE5	13.7 ± 0.6^c^	11	CD2	22.4 ± 0.7^f,g,h^
12	AK30	15.6 ± 0.3^d^	12	CIP5	22.5 ± 1.3^g,h,i^
13	AZM15	16.5 ± 0.5^d,e^	13	IPM10	25.1 ± 0.2^h,i,j^
14	GEN10	17.4 ± 0.2^e^	14	AZM15	26.1 ± 0.8^i,j^
15	COT25	24.4 ± 0.1^f^	15	LE5	26.1 ± 0.1^i,j^
16	IPM10	28.0 ± 0.5^g^	16	TE30	27.0 ± 0.3^j^

a Values are expressed as mean ± SD (*n* = 3); same lowercase letters indicate no statistically significant differences among groups according to one-way ANOVA followed by Tukey's HSD test at *p* < 0.05; all comparisons and letter assignments were performed separately within each bacterial species, and the lettering order starts from the lowest mean value to the highest.

To verify the significance of these findings and systematically interpret the observed response patterns, the data were subjected to a detailed statistical analysis. The two-way ANOVA (Table S2) showed that antibiotic efficacy was significantly influenced by both the type of antibiotic and the bacterial species. The antibiotic factor exerted a strong effect on the mean inhibition zone diameter (*F*_(15,64)_ = 369.49, *p* < 0.001, *η*^2^ = 0.989), reflecting substantial variability in activity among the tested agents. The bacterial species factor demonstrated an even greater effect (*F*_(1,64)_ = 2381.69, *p* < 0.001, *η*^2^ = 0.974), indicating a clear difference in susceptibility between the two strains. A highly significant interaction between the two factors (*F*_(15,64)_ = 170.30, *p* < 0.001, *η*^2^ = 0.976), further revealed that antibiotic efficacy was not uniform across bacterial species but depended on the structural and functional characteristics of each strain. The statistical model exhibited a very high explanatory power for the total variance (*R*_adj_^2^ = 0.991), and the assumption of homogeneity of variances was met, supporting the robustness and reliability of the results.

The one-way ANOVA revealed highly significant differences in the inhibitory performance of the tested antibiotics against *E. coli* (*F*_(15,32)_ = 1268.13, *p* < 0.001, *η*^2^ = 0.998) (Table S3) and *S. aureus* (*F*_(15,32)_ = 131.44, *p* < 0.001, *η*^2^ = 0.984) µs (Table S6), confirming that antibiotic type was the primary factor driving the variation in inhibition zones. Tukey's HSD pairwise comparisons (Tables S4 and S7) further demonstrated extensive significant contrasts among most antibiotic pairs, indicating a clear gradient in antibacterial activity and substantial differences in bacterial susceptibility.

This pattern was further supported by Tukey's HSD homogeneous subsets; for *E. coli* (Table S5), the homogeneous subset analysis identified seven statistically distinct activity subsets. Eight antibiotics showed no measurable inhibition (0 mm), while TOB10 and DOX30 exhibited limited activity (10.47 and 11.47 mm). Five antibiotics clustered within a moderate to high inhibition range (13.67–24.37 mm), and IPM10 produced the largest inhibition zone (27.97 mm), forming a uniquely superior subset. These patterns are also illustrated in Fig. S3.

Similarly, for *S. aureus* (Table S8), ten statistically distinct activity subsets were identified. CPM30 and AMP10 showed no inhibitory effect, whereas AMC20 and COT25 demonstrated low to moderate activity (11.23 and 14.27 mm). Eight antibiotics fell within a moderate to high inhibition range (17.07–22.50 mm), and the strongest inhibitory responses were recorded for IPM10, AZM15, LE5, and TE30 (25.13–26.97 mm). These patterns are also illustrated in Fig. S4.

Collectively, these findings reveal a well-defined gradient in antimicrobial efficacy across both bacterial species, underscoring pronounced differences in susceptibility profiles and resistance patterns depending on the antibiotic employed.

These findings demonstrate that the efficacy of antibiotics is fundamentally shaped by the structural differences between Gram-positive and Gram-negative bacteria, including variations in cell wall architecture, membrane permeability, efflux pump activity, and the presence of antibiotic-degrading enzymes.^67–69^ They further underscore the importance of relying on laboratory-based susceptibility testing and regularly updating treatment protocols in accordance with current local resistance patterns, particularly in light of the rising prevalence of antimicrobial resistance and the diminishing effectiveness of many conventional antibiotics.

#### 3.4.2 Susceptibility of tested pathogenic bacteria to hexane extracts

The disk diffusion assay revealed that the hexane extract of the aerial parts of *P. europaea* L. demonstrated significant antibacterial activity against the tested pathogenic bacteria, as indicated in Table 5 and Fig. S5. Notably, the flower extract exhibited a stronger inhibitory effect than that of the leave extract. Furthermore, the extracts showed markedly higher efficacy against *S. aureus* compared to *E. coli*.

**Table 5:** Mean diameters of bacterial growth inhibition zones (mm) induced by the hexane extract of the aerial parts of *P. europaea* L^a^

Pathogenic bacteria	Aerial parts	Conc. (mg per disk)								
		Negative control	20	10	5	2.5	1.25	0.625	0.312	0.156
*E. coli*	Flowers	NI	21.2 ± 0.1^a^	19.3 ± 0.3^b^	15.11 ± 0.1^c^	13.3 ± 0.2^d^	0.00^e^	NT	NT	NT
	Leaves	NI	14.2 ± 0.2^a^	12.1 ± 0.1^b^	9.5 ± 0.2^c^	0.00^d^	0.00^e^	NT	NT	NT
*S. aureus*	Flowers	NI	>40^a^	>40^a^	35.8 ± 0.3^b^	33.2 ± 0.3^c^	26.2 ± 0.2^d^	22.14 ± 0.1^e^	15.2 ± 0.1^f^	12.0 ± 0.2^g^
	Leaves	NI	29.8 ± 0.2^a^	27.5 ± 0.1^b^	23.0 ± 0.1^c^	20.6 ± 0.3^d^	18.5 ± 0.2^e^	NT	NT	NT

a NI: no inhibition.; NT: not tested.; values are expressed as mean ± SD (*n* = 3). Same lowercase letters indicate no statistically significant differences among groups for each plant part and each bacterial species separately, according to one-way ANOVA followed by Tukey's HSD test at *p* < 0.05.

The three-way ANOVA (Table S9) showed that the three factors (extract concentration, plant part, and bacterial species) constitute the primary sources of variation in the mean inhibition zone diameters. All main effects were highly significant (*P* < 0.001) and were associated with large effect sizes. Both extract concentration and bacterial species exhibited nearly complete effect size (*η*^2^ = 0.999) s, indicating that differences in concentration levels and in bacterial susceptibility account for the vast majority of the observed variation. The plant part also demonstrated a strong effect (*η*^2^ = 0.997), reflecting the chemical variability among extracts derived from different plant tissues. Significant two-way and three-way interactions with large effect sizes were also detected, suggesting that the influence of concentration does not operate independently of plant part or bacterial species, and that the overall response results from a complex interplay among these factors. Despite the strength of these interactions, their effects remained smaller than those of the main factors, indicating that the primary drivers of variation in inhibitory activity are the main effects themselves. These findings support the need for subsequent post-hoc analyses to examine differences among concentration levels within each subgroup. The model explained nearly all of the variance (*R*_adj_^2^ = 0.999), and homogeneity of variance was confirmed (*P* = 0.807), reinforcing the reliability of the results and indicating that the statistical significance reflects genuinely strong underlying effects.

The one-way ANOVA results (Tables S10, S13, S16, and S19) showed statistically significant differences among concentrations within each plant part and bacterial species combination, with very large effect sizes (*η*^2^ ≥ 0.997) and a distinct dose–response pattern when the effect of concentration within each plant part and bacterial species was examined separately. In both flower and leave extracts, inhibition zones against *E. coli* and *S. aureus* increased steadily and gradually with increasing concentrations.

These results were supported by Tukey's HSD pairwise comparisons (Tables S11, S14, S17, and S20), showing highly significant differences among most concentrations (*P* < 0.001) and revealed a steady rising trend in mean inhibition diameters. This pattern was further confirmed by Tukey's HSD homogeneous Subset analyses (Tables S12, S15, S18, and S21), as concentrations formed distinct, non-overlapping subsets, reflecting the strong biological impact of concentration. Fig. S6 and S7 illustrated a clear dose dependent trend in both plant parts against both bacterial species, with a marked superiority of the flower extract across all concentrations.

Additionally, this superiority was confirmed by direct comparisons between flower and leave extracts using independent-samples *t*-test (Tables S22 and S23). The flower extract showed significantly larger inhibition means against *E. coli* and *S. aureus*, along with highly significant differences (*P* < 0.001) and large *t*-values. Furthermore, the remarkably high effect size measures (Cohen's *d*, Hedges' *g*, and Glass's *Δ*) further suggest that this advantage represents a significant and biologically significant difference in antibacterial efficacy.

The biological differences observed are consistent with the chemical profiles of the two extracts. The flower extract contained higher proportions of terpenoid compounds (36.8% compared with 28.13% in the leaves) and fatty acid derivatives (26.31% compared with 24.3% in the leaves), both of which are widely recognized for their ability to penetrate cellular membranes, disrupt membrane permeability, and contribute to synergistic antimicrobial effects.^70–72^ Although the overall proportion of compounds previously reported to possess antibacterial activity was comparable between the two extracts (72.18% in flowers and 73.51% in leaves; Table S1), the qualitative and quantitative distribution of these compound classes provides a compelling explanation for the superior bioactivity of the flower extract.

Furthermore, *S. aureus* exhibited markedly greater sensitivity to the extracts than *E. coli*, as evidenced by the mean inhibition zones and the trends illustrated in Fig. S6 and S7, along with the significant main effect of the bacterial species factor in the three-way ANOVA analyses (Table S9). This pattern aligns with the well-established structural differences between Gram-positive and Gram-negative bacteria, the more permeable peptidoglycan-rich cell wall of Gram-positive bacteria facilitates the penetration of hydrophobic compounds, whereas the outer membrane of Gram-negative bacteria provides an additional barrier that reduces susceptibility.

The flower extract exhibited a pronounced biological superiority compared to standard antibiotics against the tested bacterial strains. One-way ANOVA (Tables S24 and S27) revealed a significant effect of both extract concentrations and antibiotics on the growth of *E. coli* and *S. aureus*, with highly significant differences (*F*_(20,42)_ = 1339.28, *P* < 0.001, *η*^2^ = 0.998) and (*F*_(23,48)_ = 308.08, *P* < 0.001, *η*^2^ = 0.994), respectively. The assumption of homogeneity of variances was satisfied, reinforcing the reliability of the statistical model and indicating that most of the variance in response was attributable to the treatment type.

Tukey's HSD pairwise comparisons showed substantial significant differences among the various extract concentrations and antibiotics (*P* < 0.001), with a clear dose–response pattern reflecting a progressive increase in antimicrobial activity. Homogeneous group analyses further supported this trend: in *E. coli* (Table S26 and Fig. S8), low concentrations exhibited limited activity, whereas the 5 mg mL^−1^ concentration surpassed antibiotics such as LE5 and DOX30. Higher concentrations (10–20 mg mL^−1^) outperformed widely used antibiotics such as GEN10, AZM15, and AK30, and approached the activity of potent antibiotics including COT25 and IPM10.

Similarly, *S. aureus* demonstrated high sensitivity to the extract components (Table S29 and Fig. S9); low concentrations (0.156–0.312 mg mL^−1^) were comparable to weak antibiotics such as AMC20 and COT25, while 0.625 mg mL^−1^ exceeded the activity of strong antibiotics including AK30, TOB10, and CN30. The intermediate concentration (1.25 mg mL^−1^) showed activity comparable to high-potency antibiotics such as TE30, AZM15, and IPM10, whereas higher concentrations (2.5–20 mg mL^−1^) clearly surpassed all tested antibiotics.

Collectively, these findings indicate that the hexane extract (particularly the flower extract) possesses strong inhibitory activity that increases with concentration, progressively surpassing weak and moderate antibiotics and ultimately matching or exceeding the efficacy of high-performance antibiotics. This highlights its promising biological potential as a candidate for developing antimicrobial agents amid the escalating challenges posed by bacterial resistance to conventional therapies.

## 4. Conclusions

This research provides the inaugural chemical and biological assessment of the hexane extract derived from the aerial parts of the Syrian plant *P. europaea* L. The extract's chemical makeup was elucidated *via* initial phytochemical screening and HS/GC-MS analysis, which indicated a prevalence of lipid-based substances, aligning with hexane's non-polar extraction properties. The lipid profile was distinguished by the identification of steroids, terpenoids, fatty acids and their esters, with fatty acid amides constituting a significant portion. Furthermore, phenolic compounds were detected. The presence of these compounds, recognized for their defensive functions in plants, implies the plant's potential exposure to insect predation or environmental pressures.

Consequently, the present investigation revealed a demonstrable antibacterial effect of the extract against the bacterial strains under examination, with the flower extract exhibiting significantly greater effectiveness compared to the leave extract, notwithstanding their shared chemical characteristics. This enhanced efficacy is ascribed to variations in the qualitative and quantitative distribution of the active constituents. Moreover, the findings indicated a discernible dose–response relationship, wherein the inhibitory activity intensified progressively with escalating concentrations, thereby substantiating the correlation between the extract's chemical makeup and the magnitude of its biological impact. Furthermore, the findings showed variation in bacterial susceptibility, *S. aureus* was more responsive to the extract than *E. coli*, consistent with the structural differences between Gram-positive and Gram-negative bacteria. In addition, comparison with standard antibiotics indicated that certain concentrations of the flower extract exhibited activity comparable to, or even exceeding, that of some antibiotics, underscoring its biological value.

The results underscore the necessity of more sophisticated chemical and biological investigations to identify and define the precise compounds responsible for the observed effects. These studies could facilitate the exploration of *P. europaea* L.'s therapeutic and nutritional potential *via* toxicity assessment, extract fractionation, compound isolation, and bioactivity evaluation, thereby potentially leading to the development of new pharmaceutical agents or functional applications.

## Author contributions

Muhannad Hasan: conceptualization, methodology, investigation, data curation, formal analysis, writing – original draft. Nidal Hasan: methodology, data curation, writing review & editing. Nathalie Moussa: chemical analysis, writing review & editing. Imad Hwija: validation, writing review & editing, supervision. Yaseer Mossa: validation, writing review & editing, supervision. Abdel Nasser Singab: chemical analysis, validation, supervision.

## Conflicts of interest

The authors declare no conflicts of interest.

## Supplementary Material

RA-016-D5RA07370G-s001

## Data Availability

The data supporting this article have been included as part of the supplementary information (SI). Supplementary information is available. See DOI: https://doi.org/10.1039/d5ra07370g.
