# Supplementary material for: Hexane extract of Plumbago europaea L. aerial parts: phytochemical screening and antibacterial activity
Source: RSC Adv. 2026 Apr 10;16(21):18921–30. doi: 10.1039/d5ra07370g (PMC13067104; doi:10.1039/d5ra07370g)
Supplement: RA-016-D5RA07370G-s001 [file RA-016-D5RA07370G-s001.pdf]

Supplementary Information (SI) for RSC Advances.
This journal is © The Royal Society of Chemistry 2026

ARTICLE

Hexane extract of *Plumbago europaea L.* aerial parts: phytochemical screening and antibacterial activity

Muhannad Hasan,*[a,d] Nidal Hasan,[b] Natalie Moussa,[c] Imad Hwija,[d] Yaseer Mossa[d] and Abdel Nasser Singab[e]

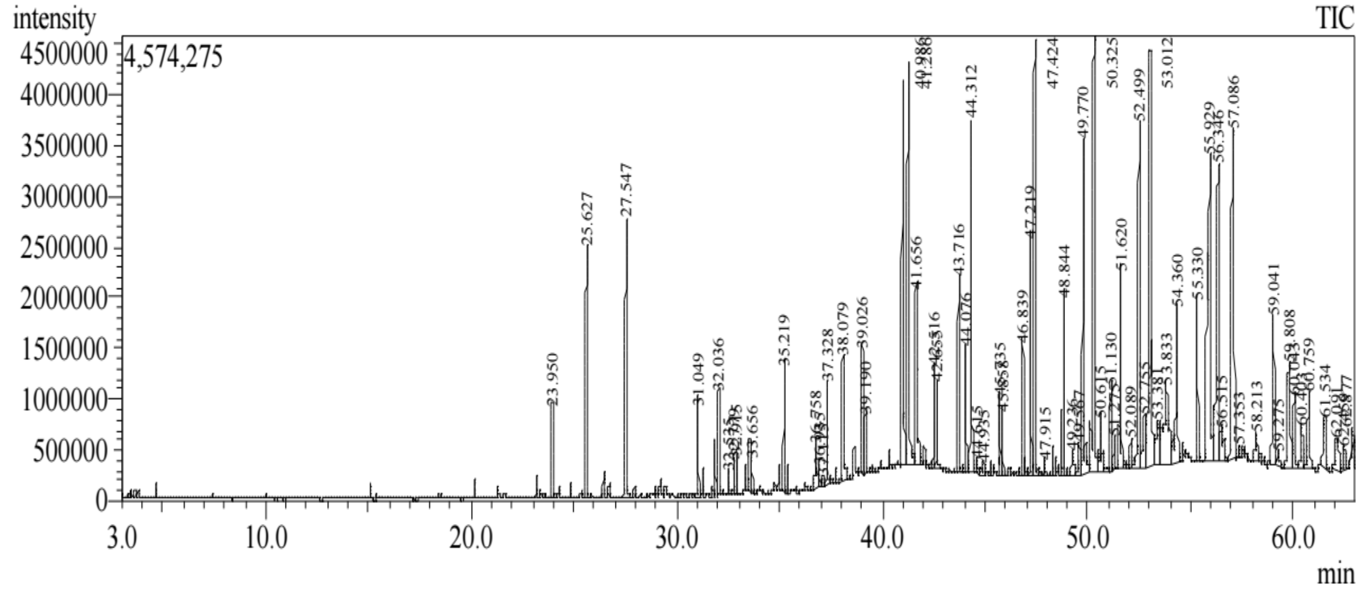

Fig. S1. The total ion chromatogram of the hexane extract of ***P. europaea L.*** flowers by HS/GC-MS.

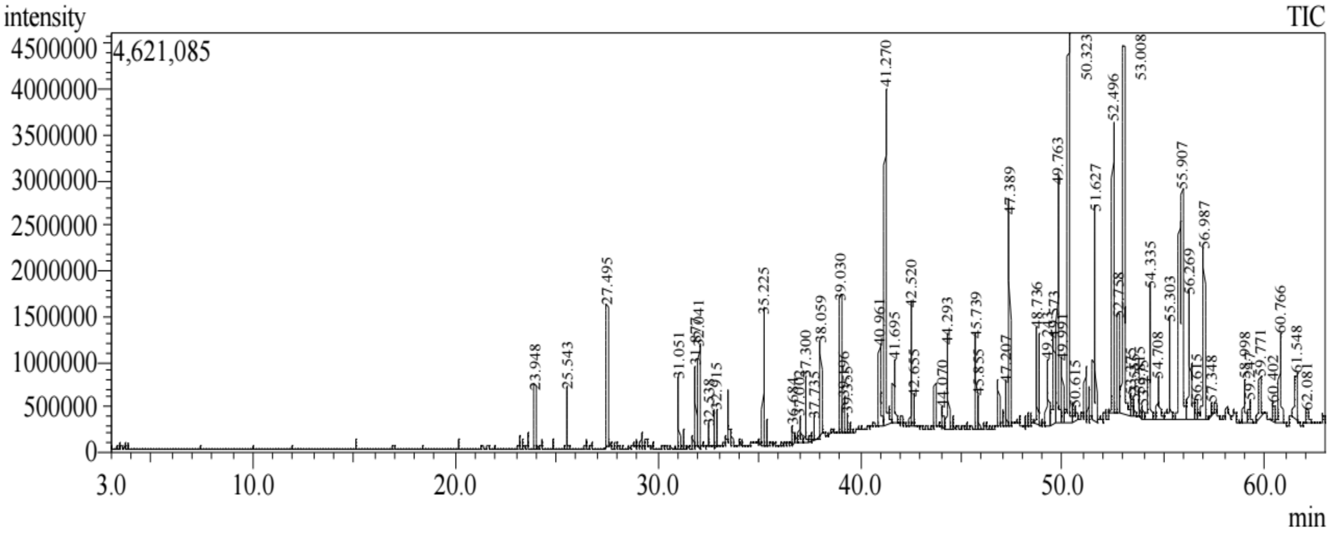

Fig. S2. The total ion chromatogram of the hexane extract of ***P. europaea L.*** leaves by HS/GC-MS.

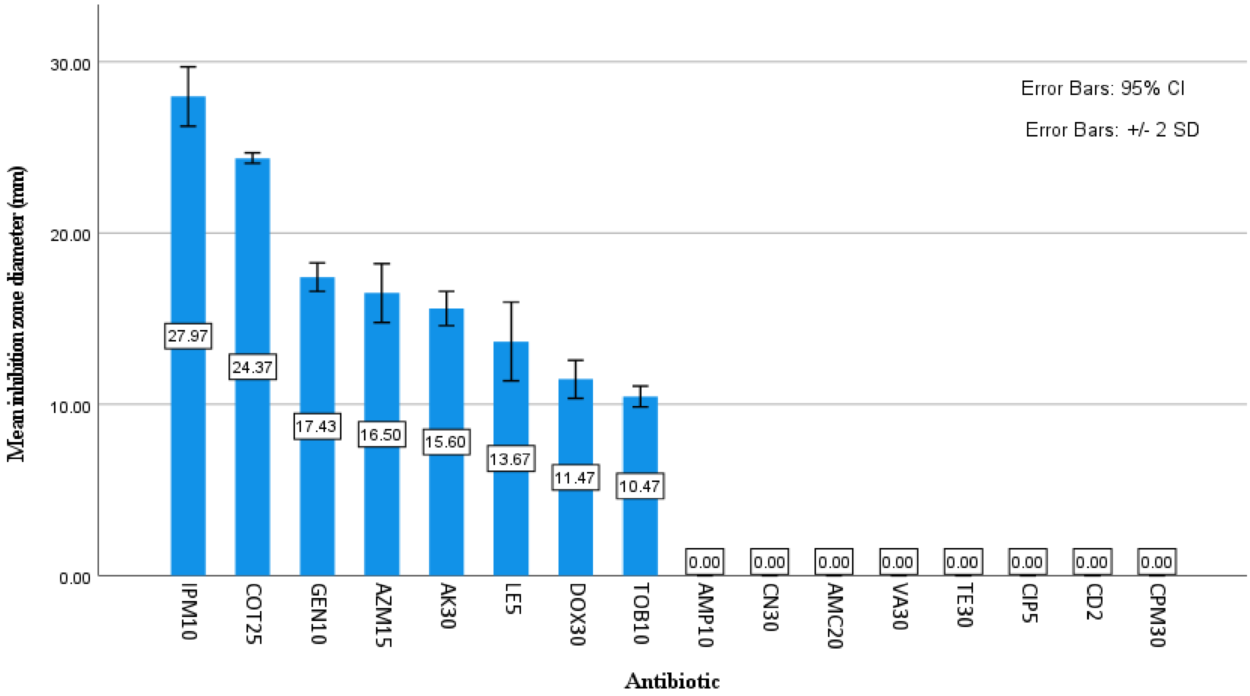

Fig. S3. The effect of active tested antibiotics on the mean inhibition zone diameters against ***E. coli***

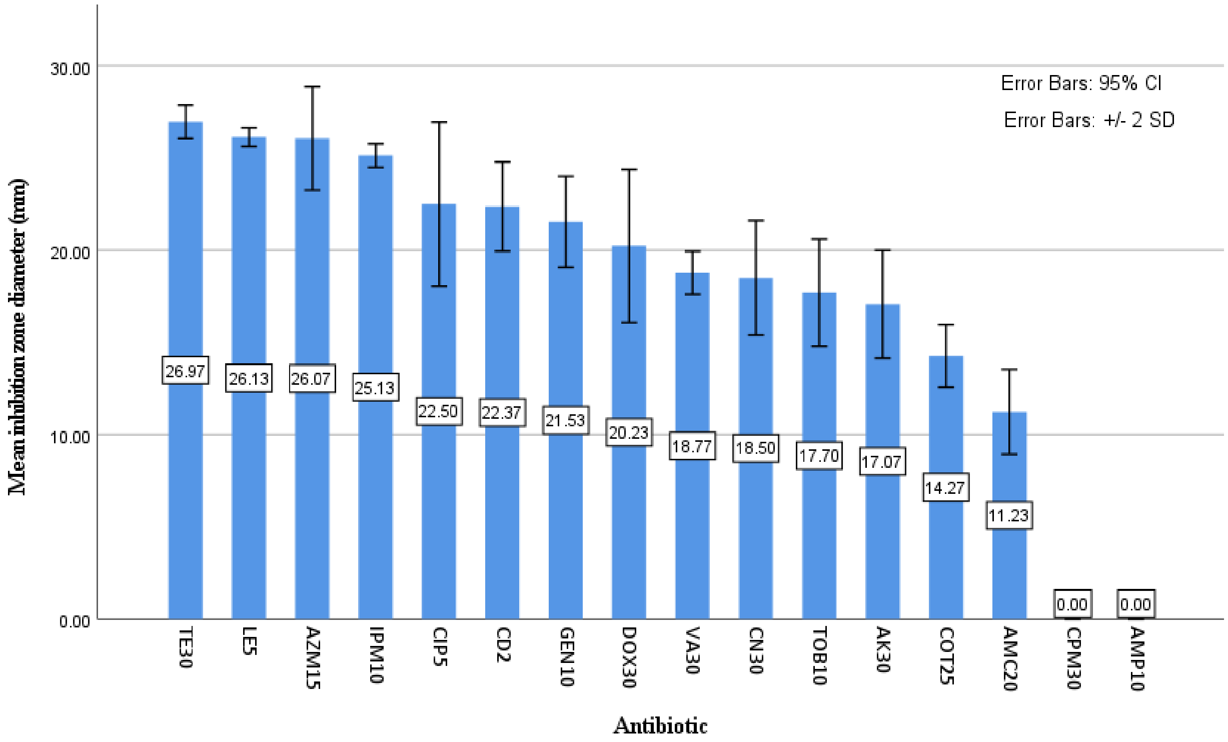

 This journal is © The Royal Society of Chemistry 20xx

Please do not adjust margins

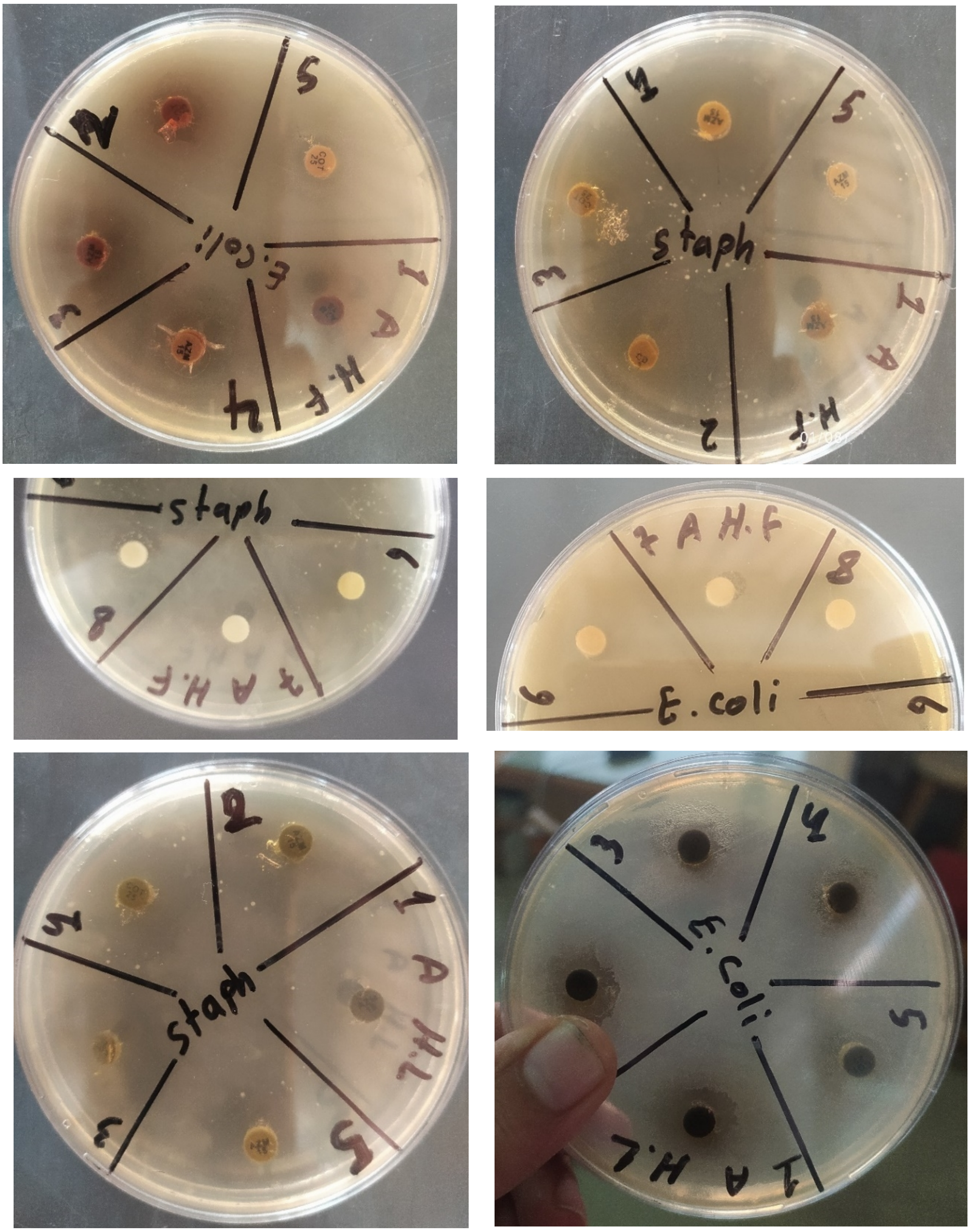

Fig. S5. Inhibitory potential of hexane extract concentrations from the aerial parts of ***P. europaea L***. against the tested pathogenic bacteria

This journal is © The Royal Society of Chemistry 20xx

Please do not adjust margins

Please do not adjust margins

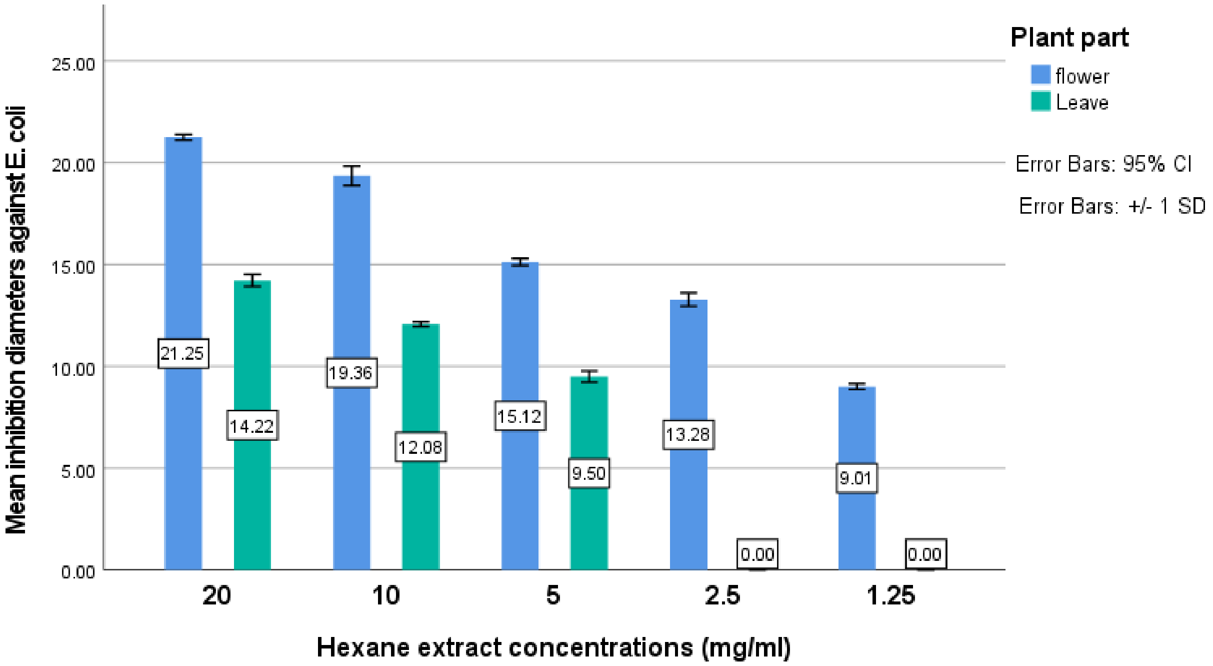

Fig. S6. Comparison of mean inhibition zone diameters of flower and leave hexane extract concentrations against against ***E. coli***

This journal is © The Royal Society of Chemistry 20xx

Please do not adjust margins

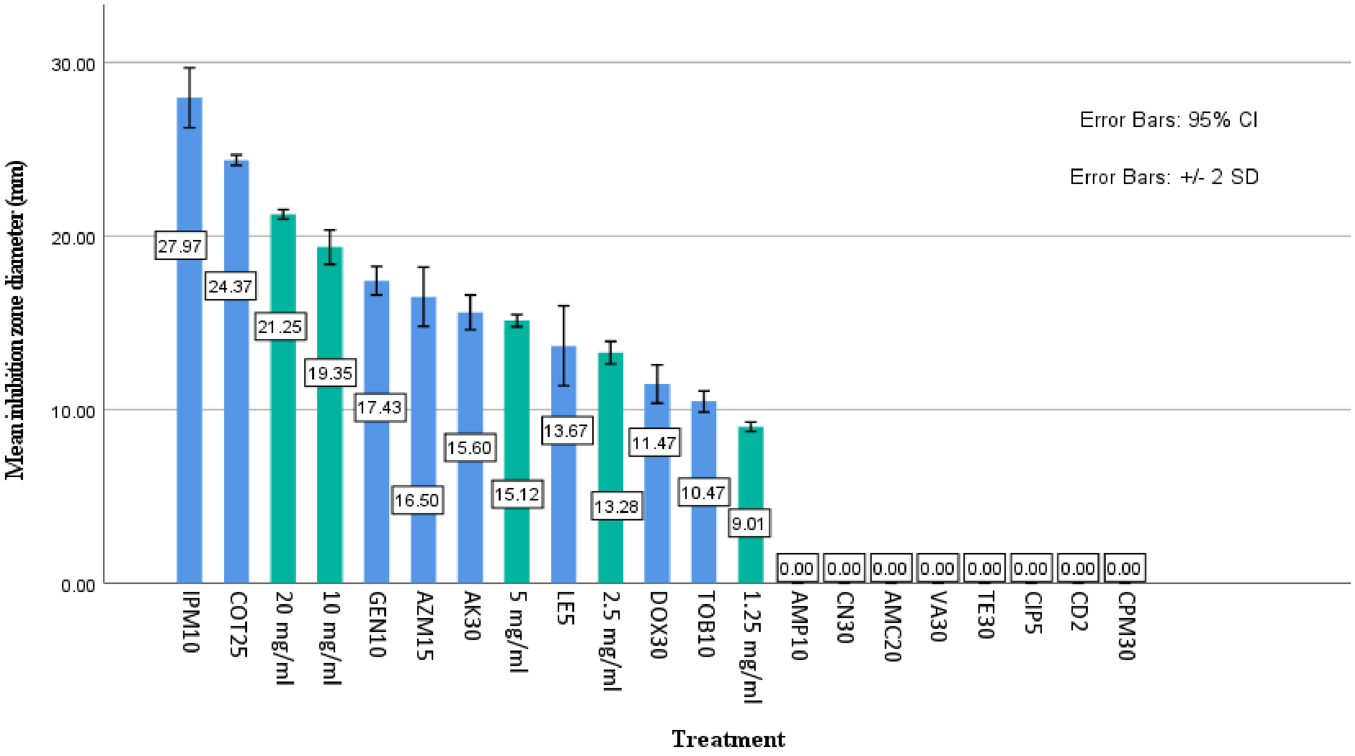
Mean inhibition zone diameter (mm)
30.00
20.00
10.00
0.00
Error Bars: 95% CI
Error Bars: +/- 2 SD
27.97
24.37
21.25
19.35
17.43
16.50
15.60
15.12
13.67
13.28
11.47
10.47
9.01
0.00
IPM10
COT25
20 mg/ml
10 mg/ml
GEN10
AZM15
AK30
5 mg/ml
LE5
2.5 mg/ml
DOX30
TOB10
1.25 mg/ml
AMP10
CN30
AMC20
VA30
TE30
CIP5
CD2
CPM30
Treatment

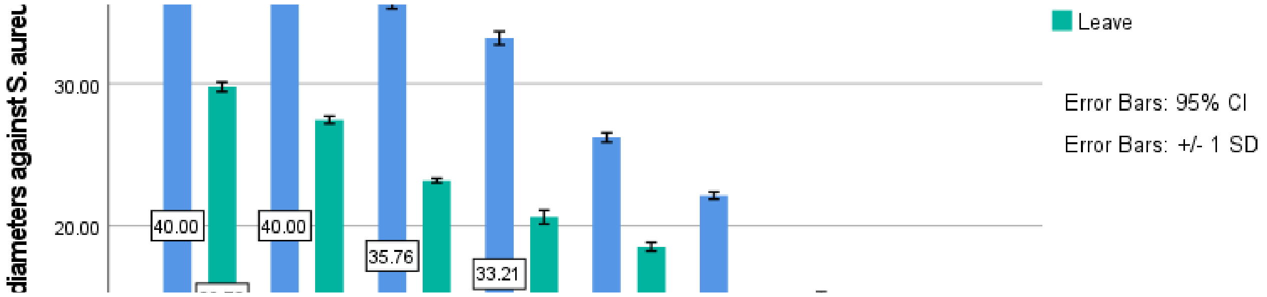
diameters against S. aureu
30.00
20.00
Leave
Error Bars: 95% CI
Error Bars: +/- 1 SD
40.00
40.00
35.76
33.21

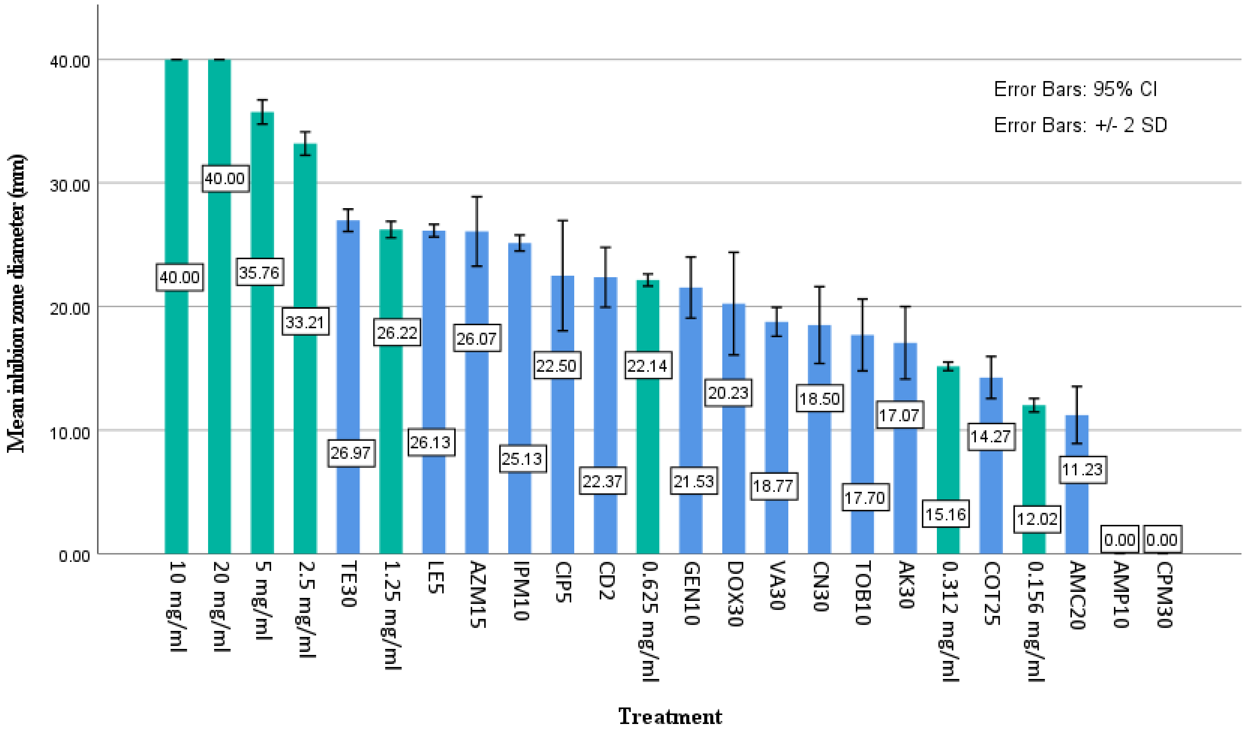
Mean inhibition zone diameter (mm)
40.00
30.00
20.00
10.00
0.00
Error Bars: 95% CI
Error Bars: +/- 2 SD
40.00
40.00
35.76
33.21
26.97
26.22
26.13
26.07
25.13
22.50
22.37
22.14
21.53
20.23
18.77
18.50
17.70
17.07
15.16
14.27
12.02
11.23
0.00
0.00
10 mg/ml
20 mg/ml
5 mg/ml
2.5 mg/ml
TE30
1.25 mg/ml
LE5
AZM15
IPM10
CIP5
CD2
0.625 mg/ml
GEN10
DOX30
VA30
CN30
TOB10
AK30
0.312 mg/ml
COT25
0.156 mg/ml
AMC20
AMP10
CPM30
Treatment

Please do not adjust margins

Fig. S9. Comparison of mean inhibition zone diameters of flower extract concentrations and antibiotics against ***S. aureus***

This journal is © The Royal Society of Chemistry 20xx

Please do not adjust margins

Table S1 Phytochemical profile of the hexane extract of aerial parts of ***P. europaea L.***

No	Name	[a]LRI	[b]LRI	M.F.	M.W.	Flower	Leave	Class	Ref.
						% ± SD			
1	4,4,6-trimethyl-cyclohex-2-en-1-ol	1085	1080	$C_9H_{16}O$	140	0.56 ∓ 0.0	0.63 ∓ 0.0	O	-
2	citronellol	1179	1169	$C_{10}H_{20}O$	156	2.99 ∓ 0.2	0.73 ∓ 0.1	M	1
3	5,9-dimethyl decan-2-one	1222	1232	$C_{12}H_{24}O$	184	3.39 ∓ 0.3	2.20 ∓ 0.2	M	-
4	3-methyl-6-propyl-Phenol	1326	1335	$C_{10}H_{14}O$	150	0.55 ∓ 0.0	0.67 ∓ 0.1	O	2
5	1-(prop-2-ynyloxy)-3,4-methylenedioxybenzene	1403	1391	$C_{10}H_{10}O_3$	178	-	0.76 ∓ 0.0	O	-
6	2-sec-butylphenol, acetate	1408	1401	$C_{12}H_{16}O_2$	192	0.59 ∓ 0.0	0.99 ∓ 0.1	O	-
7	decanamide	1425	1426	$C_{10}H_{21}NO$	171	0.19 ∓ 0.0	0.28 ∓ 0.0	FA	3
8	globulol	1530	1534	$C_{15}H_{26}O$	222	0.76 ∓ 0.1	1.34 ∓ 0.2	S	4
9	(-)-isolongifolol, methyl ether	1568	1577	$C_{16}H_{28}O$	236	0.47 ∓ 0.0	0.36 ∓ 0.0	S	-
10	tridecanoic acid, methyl ester	1580	1581	$C_{14}H_{28}O_2$	228	0.17 ∓ 0.0	0.56 ∓ 0.1	F	5
11	γ-dodecalactone	1582	1587	$C_{12}H_{22}O_2$	198	0.06 ∓ 0.0	-	O	6
12	pogostol	1601	1593	$C_{15}H_{26}O$	222	0.90 ∓ 0.1	0.83 ∓ 0.1	S	7
13	3-(1,5-dimethyl-hex-4-enyl)-2,2-dimethyl-cyclopent-3-enol	1611	1610	$C_{15}H_{26}O$	222	-	0.46 ∓ 0.1	S	-
14	junenol	1625	1635	$C_{15}H_{26}O$	222	1.16 ∓ 0.1	1.14 ∓ 0.1	S	8
15	pentadecanal	1701	1701	$C_{15}H_{30}O$	226	0.69 ∓ 0.0	1.40 ∓ 0.2	O	9
16	heptadecane	1711	1709	$C_{17}H_{36}$	240	0.41 ∓ 0.0	0.44 ∓ 0.0	H	10
17	8,14-cedrandiol	1786	1789	$C_{15}H_{26}O_2$	238	3.22 ∓ 0.3	1.35 ∓ 0.3	S	11
18	plumbagin	1801	1805	$C_{11}H_8O_3$	188	5.67 ∓ 0.4	7.75 ∓ 0.3	O	12
19	tetradecanamide	1822	1833	$C_{14}H_{29}NO$	227	1.73 ∓ 0.2	1.17 ∓ 0.1	FA	13
20	nonadecene	1900	1901	$C_{19}H_{38}$	266	0.65 ∓ 0.1	1.31 ∓ 0.1	H	14
21	(R)-(-)-14-methyl-8-hexadecyn-1-ol	1907	1911	$C_{17}H_{32}O$	252	0.52 ∓ 0.0	0.35 ∓ 0.0	O	15
22	hexadecanoic acid, ethyl ester	1978	1988	$C_{18}H_{36}O_2$	284	1.17 ∓ 0.1	-	F	16
23	eicosane	2009	2009	$C_{20}H_{42}$	282	0.96 ∓ 0.1	0.33 ∓ 0.0	H	17
24	hexadecanamide	2021	2020	$C_{16}H_{33}NO$	255	2.79 ∓ 0.2	1.52 ∓ 0.2	FA	18
25	phytol	2045	2050	$C_{20}H_{40}O$	296	0.13 ∓ 0.0	-	D	19
26	(E)-9-octadecenoic acid, methyl ester	2085	2088	$C_{19}H_{36}O_2$	296	0.58 ∓ 0.0	1.03 ∓ 0.2	F	20
27	(E,E)-9,12-octadecadienoic acid, methyl ester	2093	2093	$C_{19}H_{34}O_2$	294	0.39 ∓ 0.0	0.32 ∓ 0.0	F	21
28	cyclopentanetridecanoic acid, methyl ester	2120	2131	$C_{19}H_{36}O_2$	296	0.83 ∓ 0.1	-	F	-
29	5-(7a-isopropenyl-4,5-dimethyl-octahydroinden-4-yl)-3-methyl-pent-2-en-1-ol	2141	2146	$C_{20}H_{34}O$	290	1.80 ∓ 0.3	0.55 ∓ 0.0	D	22
30	nonadecanol	2153	2154	$C_{19}H_{40}O$	284	4.02 ∓ 0.4	2.42 ∓ 0.1	FC	23
31	9-octadecenoic acid	2175	2172	$C_{20}H_{40}O_2$	282	0.15 ∓ 0.0	-	F	24
32	eicosanal	2198	2206	$C_{20}H_{40}O$	296	-	2.07 ∓ 0.1	O	25
33	octadecanamide	2220	2215	$C_{18}H_{37}NO$	283	1.48 ∓ 0.2	-	FA	26
34	phthalic acid, butyl hexyl ester	2235	2246	$C_{16}H_{28}O_2$	306	0.36 ∓ 0.0	1.58 ∓ 0.2	O	27
35	(E)-9-octadecenoic acid, propyl ester	2284	2273	$C_{21}H_{40}O_2$	324	0.43 ∓ 0.1	2.20 ∓ 0.2	F	-
36	kolavenol acetate	2290	2289	$C_{22}H_{36}O_2$	332	2.91 ∓ 0.2	3.27 ∓ 0.3	D	-
37	nonadecanamide	2319	2308	$C_{19}H_{39}NO$	297	-	0.84 ∓ 0.1	FA	-
38	heneicosanol	2351	2341	$C_{21}H_{44}O$	312	6.81 ∓ 0.3	9.00 ∓ 0.4	FC	28
39	phthalic acid, butyl 2-ethylhexyl ester	2370	2369	$C_{20}H_{30}O_4$	334	0.53 ∓ 0.0	0.53 ∓ 0.1	O	27
40	2-methyltetracosane	2442	2433	$C_{25}H_{52}$	352	0.21 ∓ 0.0	-	H	29
41	docosanol	2451	2466	$C_{22}H_{46}O$	326	1.62 ∓ 0.1	2.88 ∓ 0.2	FC	30
42	3-cyclopentylpropionic acid, pentadecyl ester	2518	2511	$C_{23}H_{44}O_2$	352	0.34 ∓ 0.0	-	F	-
43	stigmasta-3,5-diene	2525	2533	$C_{29}H_{48}$	396	3.29 ∓ 0.2	4.20 ∓ 0.3	ST	31
44	docosyl propyl ether	2582	2576	$C_{25}H_{52}O$	368	0.65 ∓ 0.1	1.36 ∓ 0.1	O	-
45	campesterol	2632	2623	$C_{28}H_{48}O$	400	7.31 ∓ 0.4	10.26 ∓ 0.4	ST	32
46	24-noroleana-3,12-diene	2635	2634	$C_{29}H_{46}$	394	0.91 ∓ 0.1	0.43 ∓ 0.1	ST	33
47	2-methylhexacosane	2641	2650	$C_{27}H_{56}$	380	-	0.23 ∓ 0.0	H	34
48	tetracosanol	2650	2660	$C_{24}H_{50}O$	354	2.62 ∓ 0.2	0.32 ∓ 0.1	FC	28
49	(Z,Z)-9,12-octadecadienoic acid, 2,3-dihydroxypropyl ester	2697	2688	$C_{21}H_{38}O_4$	354	-	0.12 ∓ 0.0	F	-
50	β-sitosterol	2731	2727	$C_{29}H_{50}O$	414	1.94 ∓ 0.1	2.52 ∓ 0.2	ST	35
51	tetracosyl acetate	2773	2766	$C_{26}H_{52}O_2$	396	-	1.11 ∓ 0.1	F	-

This journal is © The Royal Society of Chemistry 20xx

Please do not adjust margins

52	octacosane	2804	2813	$C_{28}H_{58}$	394	1.33 ∓ 0.1	1.75 ∓ 0.2	H	36
53	β-amyrone	2869	2860	$C_{30}H_{48}O$	424	5.77 ∓ 0.3	8.33 ∓ 0.3	T	37
54	β-amyrin	2886	2887	$C_{30}H_{50}O$	426	5.67 ∓ 0.4	3.92 ∓ 0.3	T	38
55	2-methylnonacosane	2939	2930	$C_{30}H_{62}$	422	0.67 ∓ 0.1	0.32 ∓ 0.0	H	-
56	phytyl decanoate	2963	2954	$C_{30}H_{58}O_2$	450	6.13 ∓ 0.2	3.65 ∓ 0.2	D	-
57	β-amyrin acetate	3025	3027	$C_{32}H_{52}O_2$	468	0.34 ∓ 0.0	-	T	39
58	heptacosyl acetate	3071	3071	$C_{29}H_{58}O_2$	438	0.23 ∓ 0.0	0.53 ∓ 0.0	F	-
59	2-methylhentriacontane	3138	3131	$C_{32}H_{66}$	450	1.05 ∓ 0.1	-	H	-
60	phytyl dodecanoate	3162	3162	$C_{32}H_{62}O_2$	478	0.83 ∓ 0.0	-	D	-
61	octacosyl acetate	3171	3183	$C_{30}H_{60}O_2$	452	0.76 ∓ 0.0	-	F	-
62	α-tocospiro A	3195	3198	$C_{29}H_{50}O_4$	462	0.33 ∓ 0.0	-	T	40
63	3',4'-dihydrocholest-1-eno[2,1-a]naphthalene	3265	3256	$C_{35}H_{52}$	472	0.20 ∓ 0.0	-	ST	-
	Total					92.22	92.67		
	Hydrocarbons					5.28	4.38		
	Monoterpenes					6.38	2.93		
	Sesquiterpenes					6.51	5.48		
	Diterpenes					11.8	7.47		
	Triterpenoids					12.11	12.25		
	Steroids					13.65	17.77		
	Fatty amides					6.19	3.81		
	Fatty acids & Esters					5.05	5.87		
	Fatty Alcohol					15.07	14.62		
	Other oxygenated compounds					10.18	18.09		

[a]LRI: calculated retention index, [b]LRI: retention index according to Adams literature and the NIST library, M.F.: Molecular formula, M.W.: Molecule weight, % ± SD: mean ± standard deviation (n = 3), Class: Classification compound, H: Hydrocarbons, M: Monoterpenes, S: Sesquiterpenes, D: Diterpenes, T: Triterpenoids, ST: Steroids, FC: Fatty Alcohol, FA: Fatty amides, F: Fatty acids & Esters, O: Other oxygenated compounds. Ref.: references listed in this column correspond to studies reporting antibacterial activity of the compounds.

Table S2. Two-way ANOVA for the effects of antibiotic type and bacterial species on the mean inhibition zone diameter.

Source	SS	df	MS	F	P	η^2	OP
Antibiotics	5174.395	15	344.960	369.491	<0.001	0.989	1.000
Bacteria	2223.568	1	2223.568	2381.692	<0.001	0.974	1.000
Antibiotics × Bacteria	2384.901	15	158.993	170.300	<0.001	0.976	1.000
Error	59.751	64	0.934	—	—	—	—
Corrected Total	9842.615	95	—	—	—	—	—
Levene's Test (Median)	—	$df1 = 31$	$df2 = 64$	—	0.116	—	—
a. R Squared = .994 (Adjusted R Squared = .991)							

SS: Sum of Squares; **MS:** Mean Square; **η²:** partial eta squared (effect size); **OP:** Observed Power. Values represent Type III sums of squares. All effects were evaluated at α = 0.05. Levene's test (based on the median) indicated that the assumption of homogeneity of variances was satisfied. Unless otherwise stated, all ANOVA tables use the same abbreviations and statistical conventions.

Table S3. One-way ANOVA for the effect of antibiotic type on the mean inhibition zone diameter of ***E. coli.***

Source	SS	df	MS	F	P	η^2	OP
Between Groups	4326.083	15	288.406	1268.135	<0.001	0.998	1.000
Within Groups	7.278	32	0.227	—	—	—	—
Total	4333.361	47	—	—	—	—	—
Levene's Test (Median)	—	$df1 = 15$	$df2 = 9$	—	0.097	—	—

Table S4. Tukey's HSD pairwise comparisons of antibiotics on the inhibition zone diameter of ***E. coli***

Comparison		Mean Difference	Std. Error	P	95% Confidence Interval	
Anti. I	Anti. J				Lower Bound	Upper Bound
	VA30	.00000	.38938	1.000	-1.4439-	1.4439

This journal is © The Royal Society of Chemistry 20xx

Please do not adjust margins

	AMC20	.00000	.38938	1.000	-1.4439-	1.4439
	COT25	-24.36667-*	.38938	<0.001	-25.8105-	-22.9228-
	GEN10	-17.42667-*	.38938	<0.001	-18.8705-	-15.9828-
	CN30	.00000	.38938	1.000	-1.4439-	1.4439
	LE5	-13.66667-*	.38938	<0.001	-15.1105-	-12.2228-
	IPM10	-27.96667-*	.38938	<0.001	-29.4105-	-26.5228-
	AMP10	.00000	.38938	1.000	-1.4439-	1.4439
	TOB10	-10.46667-*	.38938	<0.001	-11.9105-	-9.0228-
	CD2	.00000	.38938	1.000	-1.4439-	1.4439
	DOX30	-11.46667-*	.38938	<0.001	-12.9105-	-10.0228-
	AK30	-15.60000-*	.38938	<0.001	-17.0439-	-14.1561-
	CIP5	.00000	.38938	1.000	-1.4439-	1.4439
	AZM15	-16.50000-*	.38938	<0.001	-17.9439-	-15.0561-
	TE30	.00000	.38938	1.000	-1.4439-	1.4439
	AMC20	.00000	.38938	1.000	-1.4439-	1.4439
	COT25	-24.36667-*	.38938	<0.001	-25.8105-	-22.9228-
	GEN10	-17.42667-*	.38938	<0.001	-18.8705-	-15.9828-
	CN30	.00000	.38938	1.000	-1.4439-	1.4439
	LE5	-13.66667-*	.38938	<0.001	-15.1105-	-12.2228-
	IPM10	-27.96667-*	.38938	<0.001	-29.4105-	-26.5228-
VA30	AMP10	.00000	.38938	1.000	-1.4439-	1.4439
	TOB10	-10.46667-*	.38938	<0.001	-11.9105-	-9.0228-
	CD2	.00000	.38938	1.000	-1.4439-	1.4439
	DOX30	-11.46667-*	.38938	<0.001	-12.9105-	-10.0228-
	AK30	-15.60000-*	.38938	<0.001	-17.0439-	-14.1561-
	CIP5	.00000	.38938	1.000	-1.4439-	1.4439
	AZM15	-16.50000-*	.38938	<0.001	-17.9439-	-15.0561-
	TE30	.00000	.38938	1.000	-1.4439-	1.4439
	COT25	-24.36667-*	.38938	<0.001	-25.8105-	-22.9228-
	GEN10	-17.42667-*	.38938	<0.001	-18.8705-	-15.9828-
	CN30	.00000	.38938	1.000	-1.4439-	1.4439
	LE5	-13.66667-*	.38938	<0.001	-15.1105-	-12.2228-
	IPM10	-27.96667-*	.38938	<0.001	-29.4105-	-26.5228-
	AMP10	.00000	.38938	1.000	-1.4439-	1.4439
AMC20	TOB10	-10.46667-*	.38938	<0.001	-11.9105-	-9.0228-
	CD2	.00000	.38938	1.000	-1.4439-	1.4439
	DOX30	-11.46667-*	.38938	<0.001	-12.9105-	-10.0228-
	AK30	-15.60000-*	.38938	<0.001	-17.0439-	-14.1561-
	CIP5	.00000	.38938	1.000	-1.4439-	1.4439
	AZM15	-16.50000-*	.38938	<0.001	-17.9439-	-15.0561-
	TE30	.00000	.38938	1.000	-1.4439-	1.4439
	GEN10	6.94000*	.38938	<0.001	5.4961	8.3839
	CN30	24.36667*	.38938	<0.001	22.9228	25.8105
	LE5	10.70000*	.38938	<0.001	9.2561	12.1439
	IPM10	-3.60000-*	.38938	<0.001	-5.0439-	-2.1561-
	AMP10	24.36667*	.38938	<0.001	22.9228	25.8105
COT25	TOB10	13.90000*	.38938	<0.001	12.4561	15.3439
	CD2	24.36667*	.38938	<0.001	22.9228	25.8105
	DOX30	12.90000*	.38938	<0.001	11.4561	14.3439
	AK30	8.76667*	.38938	<0.001	7.3228	10.2105
	CIP5	24.36667*	.38938	<0.001	22.9228	25.8105
	AZM15	7.86667*	.38938	<0.001	6.4228	9.3105
	TE30	24.36667*	.38938	<0.001	22.9228	25.8105
	CN30	17.42667*	.38938	<0.001	15.9828	18.8705
	LE5	3.76000*	.38938	<0.001	2.3161	5.2039
	IPM10	-10.54000-*	.38938	<0.001	-11.9839-	-9.0961-
	AMP10	17.42667*	.38938	<0.001	15.9828	18.8705
	TOB10	6.96000*	.38938	<0.001	5.5161	8.4039
GEN10	CD2	17.42667*	.38938	<0.001	15.9828	18.8705

	DOX30	5.96000*	.38938	<0.001	4.5161	7.4039
	AK30	1.82667*	.38938	.004	.3828	3.2705
	CIP5	17.42667*	.38938	<0.001	15.9828	18.8705
	AZM15	.92667	.38938	.574	-.5172-	2.3705
	TE30	17.42667*	.38938	<0.001	15.9828	18.8705
CN30	LE5	-13.66667-*	.38938	<0.001	-15.1105-	-12.2228-
	IPM10	-27.96667-*	.38938	<0.001	-29.4105-	-26.5228-
	AMP10	.00000	.38938	1.000	-1.4439-	1.4439
	TOB10	-10.46667-*	.38938	<0.001	-11.9105-	-9.0228-
	CD2	.00000	.38938	1.000	-1.4439-	1.4439
	DOX30	-11.46667-*	.38938	<0.001	-12.9105-	-10.0228-
	AK30	-15.60000-*	.38938	<0.001	-17.0439-	-14.1561-
	CIP5	.00000	.38938	1.000	-1.4439-	1.4439
	AZM15	-16.50000-*	.38938	<0.001	-17.9439-	-15.0561-
	TE30	.00000	.38938	1.000	-1.4439-	1.4439
LE5	IPM10	-14.30000-*	.38938	<0.001	-15.7439-	-12.8561-
	AMP10	13.66667*	.38938	<0.001	12.2228	15.1105
	TOB10	3.20000*	.38938	<0.001	1.7561	4.6439
	CD2	13.66667*	.38938	<0.001	12.2228	15.1105
	DOX30	2.20000*	.38938	<0.001	.7561	3.6439
	AK30	-1.93333-*	.38938	.002	-3.3772-	-.4895-
	CIP5	13.66667*	.38938	<0.001	12.2228	15.1105
	AZM15	-2.83333-*	.38938	<0.001	-4.2772-	-1.3895-
	TE30	13.66667*	.38938	<0.001	12.2228	15.1105
IPM10	AMP10	27.96667*	.38938	<0.001	26.5228	29.4105
	TOB10	17.50000*	.38938	<0.001	16.0561	18.9439
	CD2	27.96667*	.38938	<0.001	26.5228	29.4105
	DOX30	16.50000*	.38938	<0.001	15.0561	17.9439
	AK30	12.36667*	.38938	<0.001	10.9228	13.8105
	CIP5	27.96667*	.38938	<0.001	26.5228	29.4105
	AZM15	11.46667*	.38938	<0.001	10.0228	12.9105
	TE30	27.96667*	.38938	<0.001	26.5228	29.4105
AMP10	TOB10	-10.46667-*	.38938	<0.001	-11.9105-	-9.0228-
	CD2	.00000	.38938	1.000	-1.4439-	1.4439
	DOX30	-11.46667-*	.38938	<0.001	-12.9105-	-10.0228-
	AK30	-15.60000-*	.38938	<0.001	-17.0439-	-14.1561-
	CIP5	.00000	.38938	1.000	-1.4439-	1.4439
	AZM15	-16.50000-*	.38938	<0.001	-17.9439-	-15.0561-
	TE30	.00000	.38938	1.000	-1.4439-	1.4439
TOB10	CD2	10.46667*	.38938	<0.001	9.0228	11.9105
	DOX30	-1.00000-	.38938	.452	-2.4439-	.4439
	AK30	-5.13333-*	.38938	<0.001	-6.5772-	-3.6895-
	CIP5	10.46667*	.38938	<0.001	9.0228	11.9105
	AZM15	-6.03333-*	.38938	<0.001	-7.4772-	-4.5895-
	TE30	10.46667*	.38938	<0.001	9.0228	11.9105
CD2	DOX30	-11.46667-*	.38938	<0.001	-12.9105-	-10.0228-
	AK30	-15.60000-*	.38938	<0.001	-17.0439-	-14.1561-
	CIP5	.00000	.38938	1.000	-1.4439-	1.4439
	AZM15	-16.50000-*	.38938	<0.001	-17.9439-	-15.0561-
	TE30	.00000	.38938	1.000	-1.4439-	1.4439
DOX30	AK30	-4.13333-*	.38938	<0.001	-5.5772-	-2.6895-
	CIP5	11.46667*	.38938	<0.001	10.0228	12.9105
	AZM15	-5.03333-*	.38938	<0.001	-6.4772-	-3.5895-
	TE30	11.46667*	.38938	<0.001	10.0228	12.9105
AK30	CIP5	15.60000*	.38938	<0.001	14.1561	17.0439
	AZM15	-.90000-	.38938	.619	-2.3439-	.5439
	TE30	15.60000*	.38938	<0.001	14.1561	17.0439
CIP5	AZM15	-16.50000-*	.38938	<0.001	-17.9439-	-15.0561-
	TE30	.00000	.38938	1.000	-1.4439-	1.4439

This journal is © The Royal Society of Chemistry 20xx

AZM15	TE30	16.50000*	.38938	<0.001	15.0561	17.9439

*: indicate pairwise differences that are statistically significant according to Tukey's HSD test at α = 0.05. Positive mean differences indicate higher inhibition at the first antibiotic (Anti. I) compared to the second (Anti. J). Unless otherwise stated, all Tukey HSD test comparison tables use the same interpretive conventions.

Table S5 Tukey's HSD homogeneous groups for the antibiotic effects on the inhibition zone diameter of ***E. coli***

**Antibiotic**	**N**	**Subset for $\alpha = 0.05$**						
		**1**	**2**	**3**	**4**	**5**	**6**	**7**
**CPM30**	3	0.00						
**VA30**	3	0.00						
**AMC20**	3	0.00						
**CN30**	3	0.00						
**AMP10**	3	0.00						
**CD2**	3	0.00						
**CIP5**	3	0.00						
**TE30**	3	0.00						
**TOB10**	3		10.47					
**DOX30**	3		11.47					
**LE5**	3			13.67				
**AK30**	3				15.60			
**AZM15**	3				16.50	16.50		
**GEN10**	3					17.43		
**COT25**	3						24.37	
**IPM10**	3							27.97
P		1.000	.452	1.000	.619	.574	1.000	1.000

Means represent homogeneous subsets identified by Tukey's HSD test; antibiotics within the same subset do not differ significantly in their inhibition levels. Higher mean values correspond to greater inhibition. Unless otherwise stated, all tables presenting Tukey HSD homogeneous groups use the same abbreviations and interpretive conventions.

Table S6. One-way ANOVA for the effect of antibiotic type on the mean inhibition zone diameter of ***S. aureus.***

**Source**	SS	df	MS	F	P	η^2	OP
Between Groups	3086.426	15	205.762	139.283	<0.001	0.985	1.000
Within Groups	47.273	32	1.477	—	—	—	—
Total	4333.361	47	—	—	—	—	—
Levene's Test (Median)	—	$df1 = 15$	$df2 = 16$	—	0.442	—	—

Table S7. Tukey's HSD pairwise comparisons of antibiotics on the inhibition zone diameter of ***S. aureus.***

**Comparison**		**Mean Difference**	**Std. Error**	P	**95% Confidence Interval**	
**Anti. I**	**Anti. J**				**Lower Bound**	**Upper Bound**
COT25	VA30	-18.76667-*	.99240	<0.001	-22.4466-	-15.0868-
	AMC20	-11.23333-*	.99240	<0.001	-14.9132-	-7.5534-
	COT25	-14.26667-*	.99240	<0.001	-17.9466-	-10.5868-
	GEN10	-21.53333-*	.99240	<0.001	-25.2132-	-17.8534-
	CN30	-18.50000-*	.99240	<0.001	-22.1799-	-14.8201-
	LE5	-26.13333-*	.99240	<0.001	-29.8132-	-22.4534-
	IPM10	-25.13333-*	.99240	<0.001	-28.8132-	-21.4534-
	AMP10	.00000	.99240	1.000	-3.6799-	3.6799
	TOB10	-17.70000-*	.99240	<0.001	-21.3799-	-14.0201-
	CD2	-22.36667-*	.99240	<0.001	-26.0466-	-18.6868-
	DOX30	-20.23333-*	.99240	<0.001	-23.9132-	-16.5534-
	AK30	-17.06667-*	.99240	<0.001	-20.7466-	-13.3868-
	CIP5	-22.50000-*	.99240	<0.001	-26.1799-	-18.8201-
	AZM15	-26.06667-*	.99240	<0.001	-29.7466-	-22.3868-

This journal is © The Royal Society of Chemistry 20xx

	TE30	-26.96667-*	.99240	<0.001	-30.6466-	-23.2868-
VA30	AMC20	7.53333*	.99240	<0.001	3.8534	11.2132
	COT25	4.50000*	.99240	.006	.8201	8.1799
	GEN10	-2.76667-	.99240	.324	-6.4466-	.9132
	CN30	.26667	.99240	1.000	-3.4132-	3.9466
	LE5	-7.36667-*	.99240	<0.001	-11.0466-	-3.6868-
	IPM10	-6.36667-*	.99240	<0.001	-10.0466-	-2.6868-
	AMP10	18.76667*	.99240	<0.001	15.0868	22.4466
	TOB10	1.06667	.99240	.999	-2.6132-	4.7466
	CD2	-3.60000-	.99240	.060	-7.2799-	.0799
	DOX30	-1.46667-	.99240	.977	-5.1466-	2.2132
	AK30	1.70000	.99240	.928	-1.9799-	5.3799
	CIP5	-3.73333-*	.99240	.044	-7.4132-	-.0534-
	AZM15	-7.30000-*	.99240	<0.001	-10.9799-	-3.6201-
	TE30	-8.20000-*	.99240	<0.001	-11.8799-	-4.5201-
AMC20	COT25	-3.03333-	.99240	.202	-6.7132-	.6466
	GEN10	-10.30000-*	.99240	<0.001	-13.9799-	-6.6201-
	CN30	-7.26667-*	.99240	<0.001	-10.9466-	-3.5868-
	LE5	-14.90000-*	.99240	<0.001	-18.5799-	-11.2201-
	IPM10	-13.90000-*	.99240	<0.001	-17.5799-	-10.2201-
	AMP10	11.23333*	.99240	<0.001	7.5534	14.9132
	TOB10	-6.46667-*	.99240	<0.001	-10.1466-	-2.7868-
	CD2	-11.13333-*	.99240	<0.001	-14.8132-	-7.4534-
	DOX30	-9.00000-*	.99240	<0.001	-12.6799-	-5.3201-
	AK30	-5.83333-*	.99240	<0.001	-9.5132-	-2.1534-
	CIP5	-11.26667-*	.99240	<0.001	-14.9466-	-7.5868-
	AZM15	-14.83333-*	.99240	<0.001	-18.5132-	-11.1534-
	TE30	-15.73333-*	.99240	<0.001	-19.4132-	-12.0534-
COT25	GEN10	-7.26667-*	.99240	<0.001	-10.9466-	-3.5868-
	CN30	-4.23333-*	.99240	.013	-7.9132-	-.5534-
	LE5	-11.86667-*	.99240	<0.001	-15.5466-	-8.1868-
	IPM10	-10.86667-*	.99240	<0.001	-14.5466-	-7.1868-
	AMP10	14.26667*	.99240	<0.001	10.5868	17.9466
	TOB10	-3.43333-	.99240	.088	-7.1132-	.2466
	CD2	-8.10000-*	.99240	<0.001	-11.7799-	-4.4201-
	DOX30	-5.96667-*	.99240	<0.001	-9.6466-	-2.2868-
	AK30	-2.80000-	.99240	.307	-6.4799-	.8799
	CIP5	-8.23333-*	.99240	<0.001	-11.9132-	-4.5534-
	AZM15	-11.80000-*	.99240	<0.001	-15.4799-	-8.1201-
	TE30	-12.70000-*	.99240	<0.001	-16.3799-	-9.0201-
GEN10	CN30	3.03333	.99240	.202	-.6466-	6.7132
	LE5	-4.60000-*	.99240	.005	-8.2799-	-.9201-
	IPM10	-3.60000-	.99240	.060	-7.2799-	.0799
	AMP10	21.53333*	.99240	<0.001	17.8534	25.2132
	TOB10	3.83333*	.99240	.035	.1534	7.5132
	CD2	-.83333-	.99240	1.000	-4.5132-	2.8466
	DOX30	1.30000	.99240	.992	-2.3799-	4.9799
	AK30	4.46667*	.99240	.007	.7868	8.1466
	CIP5	-.96667-	.99240	1.000	-4.6466-	2.7132
	AZM15	-4.53333-*	.99240	.006	-8.2132-	-.8534-
	TE30	-5.43333-*	.99240	<0.001	-9.1132-	-1.7534-
CN30	LE5	-7.63333-*	.99240	<0.001	-11.3132-	-3.9534-
	IPM10	-6.63333-*	.99240	<0.001	-10.3132-	-2.9534-
	AMP10	18.50000*	.99240	<0.001	14.8201	22.1799
	TOB10	.80000	.99240	1.000	-2.8799-	4.4799
	CD2	-3.86667-*	.99240	.032	-7.5466-	-.1868-
	DOX30	-1.73333-	.99240	.918	-5.4132-	1.9466
	AK30	1.43333	.99240	.982	-2.2466-	5.1132
	CIP5	-4.00000-*	.99240	.023	-7.6799-	-.3201-

This journal is © The Royal Society of Chemistry 20xx

	AZM15	-7.56667-*	.99240	<0.001	-11.2466-	-3.8868-
	TE30	-8.46667-*	.99240	<0.001	-12.1466-	-4.7868-
LE5	IPM10	1.00000	.99240	1.000	-2.6799-	4.6799
	AMP10	26.13333*	.99240	<0.001	22.4534	29.8132
	TOB10	8.43333*	.99240	<0.001	4.7534	12.1132
	CD2	3.76667*	.99240	.041	.0868	7.4466
	DOX30	5.90000*	.99240	<0.001	2.2201	9.5799
	AK30	9.06667*	.99240	<0.001	5.3868	12.7466
	CIP5	3.63333	.99240	.056	-.0466-	7.3132
	AZM15	.06667	.99240	1.000	-3.6132-	3.7466
	TE30	-.83333-	.99240	1.000	-4.5132-	2.8466
IPM10	AMP10	25.13333*	.99240	<0.001	21.4534	28.8132
	TOB10	7.43333*	.99240	<0.001	3.7534	11.1132
	CD2	2.76667	.99240	.324	-.9132-	6.4466
	DOX30	4.90000*	.99240	.002	1.2201	8.5799
	AK30	8.06667*	.99240	<0.001	4.3868	11.7466
	CIP5	2.63333	.99240	.400	-1.0466-	6.3132
	AZM15	-.93333-	.99240	1.000	-4.6132-	2.7466
	TE30	-1.83333-	.99240	.880	-5.5132-	1.8466
AMP10	TOB10	-17.70000-*	.99240	<0.001	-21.3799-	-14.0201-
	CD2	-22.36667-*	.99240	<0.001	-26.0466-	-18.6868-
	DOX30	-20.23333-*	.99240	<0.001	-23.9132-	-16.5534-
	AK30	-17.06667-*	.99240	<0.001	-20.7466-	-13.3868-
	CIP5	-22.50000-*	.99240	<0.001	-26.1799-	-18.8201-
	AZM15	-26.06667-*	.99240	<0.001	-29.7466-	-22.3868-
	TE30	-26.96667-*	.99240	<0.001	-30.6466-	-23.2868-
TOB10	CD2	-4.66667-*	.99240	.004	-8.3466-	-.9868-
	DOX30	-2.53333-	.99240	.462	-6.2132-	1.1466
	AK30	.63333	.99240	1.000	-3.0466-	4.3132
	CIP5	-4.80000-*	.99240	.003	-8.4799-	-1.1201-
	AZM15	-8.36667-*	.99240	<0.001	-12.0466-	-4.6868-
	TE30	-9.26667-*	.99240	<0.001	-12.9466-	-5.5868-
CD2	DOX30	2.13333	.99240	.722	-1.5466-	5.8132
	AK30	5.30000*	.99240	.001	1.6201	8.9799
	CIP5	-.13333-	.99240	1.000	-3.8132-	3.5466
	AZM15	-3.70000-*	.99240	.048	-7.3799-	-.0201-
	TE30	-4.60000-*	.99240	.005	-8.2799-	-.9201-
DOX30	AK30	3.16667	.99240	.155	-.5132-	6.8466
	CIP5	-2.26667-	.99240	.637	-5.9466-	1.4132
	AZM15	-5.83333-*	.99240	<0.001	-9.5132-	-2.1534-
	TE30	-6.73333-*	.99240	<0.001	-10.4132-	-3.0534-
AK30	CIP5	-5.43333-*	.99240	<0.001	-9.1132-	-1.7534-
	AZM15	-9.00000-*	.99240	<0.001	-12.6799-	-5.3201-
	TE30	-9.90000-*	.99240	<0.001	-13.5799-	-6.2201-
CIP5	AZM15	-3.56667-	.99240	.065	-7.2466-	.1132
	TE30	-4.46667-*	.99240	.007	-8.1466-	-.7868-
AZM15	TE30	-.90000-	.99240	1.000	-4.5799-	2.7799

Table S8. Tukey's HSD homogeneous groups for the antibiotic effects on the inhibition zone diameter of ***S. aureus.***

Antibiotic	N	Subset for $\alpha = 0.05$									
		1	2	3	4	5	6	7	8	9	10
**CPM30**	3	0.00	—	—	—	—	—	—	—	—	—
**AMP10**	3	0.00	—	—	—	—	—	—	—	—	—
**AMC20**	3	—	11.23	—	—	—	—	—	—	—	—
**COT25**	3	—	14.27	14.27	—	—	—	—	—	—	—
**AK30**	3	—	—	17.07	17.07	—	—	—	—	—	—

**TOB10**	3	—	—	17.70	17.70	—	—	—	—	—	—
**CN30**	3	—	—	—	18.50	18.50	—	—	—	—	—
**VA30**	3	—	—	—	18.77	18.77	18.77	—	—	—	—
**DOX30**	3	—	—	—	20.23	20.23	20.23	20.23	—	—	—
**GEN10**	3	—	—	—	—	21.53	21.53	21.53	21.53	—	—
**CD2**	3	—	—	—	—	—	22.37	22.37	22.37	—	—
**CIP5**	3	—	—	—	—	—	—	22.50	22.50	22.50	—
**IPM10**	3	—	—	—	—	—	—	—	25.13	25.13	25.13
**AZM15**	3	—	—	—	—	—	—	—	—	26.07	26.07
**LE5**	3	—	—	—	—	—	—	—	—	26.13	26.13
**TE30**	3	—	—	—	—	—	—	—	—	—	26.97
P		1.000	.202	.088	.155	.202	.060	.637	.060	.056	.880

Table S9. Three-way ANOVA for the interactive effects of concentration, plant part, and bacterial species on the mean Inhibition zone diameter.

Source	SS	df	MS	F	P	η^2	OP
Concentration	3490.555	7	498.651	6106.577	<0.001	0.999	1.000
Plant Part	1439.522	1	1439.522	17628.680	<0.001	0.997	1.000
Bacteria	4905.285	1	4905.285	60071.111	<0.001	0.999	1.000
Concentration × Plant Part	41.154	4	10.288	125.994	<0.001	0.916	1.000
Concentration × Bacteria	20.092	4	5.023	61.513	<0.001	0.842	1.000
Plant Part × Bacteria	27.486	1	27.486	336.602	<0.001	0.880	1.000
Concentration × Plant Part × Bacteria	40.298	4	10.074	123.374	<0.001	0.915	1.000
Error	3.756	46	0.082	—	—	—	—
Corrected Total	8146.811	68	—	—	—	—	—
Levene's Test (Median)	—	$df1 = 22$	$df2 = 46$	—	0.807	—	—
a. R Squared = 1.000 (Adjusted R Squared = 0.999)							

Table S10. One-way ANOVA for the effect of different concentrations of flower extract on the mean inhibition zone diameter of ***E. coli***.

Source	SS	df	MS	F	P	η^2	OP
Between Groups	285.002	4	71.250	855.142	<0.001	0.997	1.000
Within Groups	0.833	10	0.083	—	—	—	—
Total	285.835	14	—	—	—	—	—
Levene's Test (Median)	—	$df1 = 4$	$df2 = 10$	—	0.563	—	—

Table S11. Tukey's HSD pairwise comparisons of flower extract concentrations on the inhibition zone diameter of ***E. coli.***

Comparison		Mean Difference	Std. Error	P	95% Confidence Interval	
**Conc. I (mg/ml)**	**Conc. J (mg/ml)**				**Lower Bound**	**Upper Bound**
20	10	1.89667*	0.23568	<0.001	1.1210	2.6723
	5	6.12333*	0.23568	<0.001	5.3477	6.8990
	2.5	7.97000*	0.23568	<0.001	7.1943	8.7457
	1.25	12.23667*	0.23568	<0.001	11.4610	13.0123
10	5	4.22667*	0.23568	<0.001	3.4510	5.0023
	2.5	6.07333*	0.23568	<0.001	5.2977	6.8490
	1.25	10.34000*	0.23568	<0.001	9.5643	11.1157
5	2.5	1.84667*	0.23568	<0.001	1.0710	2.6223
	1.25	6.11333*	0.23568	<0.001	5.3377	6.8890
2.5	1.25	4.26667*	0.23568	<0.001	3.4910	5.0423

This journal is © The Royal Society of Chemistry 20xx

Please do not adjust margins
Please do not adjust margins

Please do not adjust margins

Table S12. Tukey's HSD homogeneous subsets for the flower extract concentrations on the inhibition zone diameter of ***E. coli***

**Conc. (mg/mL)**	**N**	**Subset for $\alpha = 0.05$**				
		**1**	**2**	**3**	**4**	**5**
**1.25**	3	9.01	—	—	—	—
**2.5**	3	—	13.28	—	—	—
**5**	3	—	—	15.12	—	—
**10**	3	—	—	—	19.35	—
**20**	3	—	—	—	—	21.25
P		1.00	1.00	1.00	1.00	1.00

Table S13. One-way ANOVA for the effect of different concentrations of leave extract on the mean inhibition zone diameter of ***E. coli***.

**Source**	SS	df	MS	F	P	η^2	OP
Between Groups	546.025	4	136.506	3763.269	<0.001	0.9993	1.000
Within Groups	0.363	10	0.036	—	—	—	—
Total	285.835	14	—	—	—	—	—
Levene's Test (Median)	—	$df1 = 4$	$df2 = 5$	—	0.274	—	—

Table S14. Tukey's HSD pairwise comparisons of leave extract concentrations on the inhibition zone diameter of ***E. coli.***

**Comparison**		**Mean Difference**	**Std. Error**	P	**95% Confidence Interval**	
**Conc. I (mg/ml)**	**Conc. J (mg/ml)**				**Lower Bound**	**Upper Bound**
20	10	2.14333*	0.15551	<0.001	1.6315	2.6551
	5	4.72333*	0.15551	<0.001	4.2115	5.2351
	2.5	14.22000*	0.15551	<0.001	13.7082	14.7318
	1.25	14.22000*	0.15551	<0.001	13.7082	14.7318
10	5	2.58000*	0.15551	<0.001	2.0682	3.0918
	2.5	12.07667*	0.15551	<0.001	11.5649	12.5885
	1.25	12.07667*	0.15551	<0.001	11.5649	12.5885
5	2.5	9.49667*	0.15551	<0.001	8.9849	10.0085
	1.25	9.49667*	0.15551	<0.001	8.9849	10.0085
2.5	1.25	0.00000	0.15551	1.000	–0.5118	0.5118

Table S15. Tukey's HSD homogeneous subsets for the leave extract concentrations on the inhibition zone diameter of ***E. coli***

**Conc. (mg/mL)**	**N**	**Subset for $\alpha = 0.05$**			
		**1**	**2**	**3**	**4**
**1.25**	3	0.00	—	—	—
**2.5**	3	0.00	—	—	—
**5**	3	—	9.50	—	—
**10**	3	—	—	12.08	—
**20**	3	—	—	—	14.22
P	—	1.00	1.00	1.00	1.00

Please do not adjust margins

Please do not adjust margins

Table S16. One-way ANOVA for the effect of different concentrations of flower extract on the mean inhibition zone diamete r of ***S. aureus***.

Source	SS	df	MS	F	P	η^2	OP
Between Groups	2498.574	7	356.939	3870.832	<0.001	0.9994	1.000
Within Groups	1.475	16	0.092	—	—	—	—
Total	2500.049	23	—	—	—	—	—
Levene's Test (Median)	—	$df1 = 7$	$df2 = 7$	—	0.688	—	—

Table S17. Tukey's HSD pairwise comparisons of flower extract concentrations on the inhibition zone diameter of ***S. aureus***.

Comparison		Mean Difference	Std. Error	P	95% Confidence Interval	
Conc. I (mg/ml)	Conc. J (mg/ml)				Lower Bound	Upper Bound
20	10	.00000	.24794	1.000	-.8584-	.8584
	5	4.24000*	.24794	.000	3.3816	5.0984
	2.5	6.79000*	.24794	.000	5.9316	7.6484
	1.25	13.77667*	.24794	.000	12.9183	14.6351
	0.625	17.86000*	.24794	.000	17.0016	18.7184
	0.312	24.83667*	.24794	.000	23.9783	25.6951
	0.156	27.97667*	.24794	.000	27.1183	28.8351
10	5	4.24000*	.24794	.000	3.3816	5.0984
	2.5	6.79000*	.24794	.000	5.9316	7.6484
	1.25	13.77667*	.24794	.000	12.9183	14.6351
	0.625	17.86000*	.24794	.000	17.0016	18.7184
	0.312	24.83667*	.24794	.000	23.9783	25.6951
	0.156	27.97667*	.24794	.000	27.1183	28.8351
5	2.5	2.55000*	.24794	.000	1.6916	3.4084
	1.25	9.53667*	.24794	.000	8.6783	10.3951
	0.625	13.62000*	.24794	.000	12.7616	14.4784
	0.312	20.59667*	.24794	.000	19.7383	21.4551
	0.156	23.73667*	.24794	.000	22.8783	24.5951
2.5	1.25	6.98667*	.24794	.000	6.1283	7.8451
	0.625	11.07000*	.24794	.000	10.2116	11.9284
	0.312	18.04667*	.24794	.000	17.1883	18.9051
	0.156	21.18667*	.24794	.000	20.3283	22.0451
1.25	0.625	4.08333*	.24794	.000	3.2249	4.9417
	0.312	11.06000*	.24794	.000	10.2016	11.9184
	0.156	14.20000*	.24794	.000	13.3416	15.0584
0.625	0.312	6.97667*	.24794	.000	6.1183	7.8351
	0.156	10.11667*	.24794	.000	9.2583	10.9751
0.312	0.156	3.14000*	.24794	.000	2.2816	3.9984

Table S18. Tukey's HSD homogeneous subsets for the flower extract concentrations on the inhibition zone diameter of ***S. aureus***.

Conc. (mg/mL)	N	Subset for $\alpha = 0.05$						
		1	2	3	4	5	6	7
**0.156**	3	12.02	—	—	—	—	—	—
**0.312**	3	—	15.16	—	—	—	—	—
**0.625**	3	—	—	22.14	—	—	—	—
**1.25**	3	—	—	—	26.22	—	—	—
**2.5**	3	—	—	—	—	33.21	—	—
**5**	3	—	—	—	—	—	35.76	—
**10**	3	—	—	—	—	—	—	40.00
**20**	3	—	—	—	—	—	—	40.00
P	—	1.00	1.00	1.00	1.00	1.00	1.00	1.00

This journal is © The Royal Society of Chemistry 20xx

Please do not adjust margins

Table S19. One-way ANOVA for the effect of different concentrations of leave extract on the mean inhibition zone diameter of ***S. aureus***.

**Source**	SS	df	MS	F	P	η^2	OP
Between Groups	262.498	4	65.624	604.871	<0.001	0.997	1.000
Within Groups	1.085	10	0.108	—	—	—	—
Total	263.583	14	—	—	—	—	—
Levene's Test (Median)	—	$df1 = 4$	$df2 = 8$	—	0.874	—	—

Table S20. Tukey's HSD pairwise comparisons of leave extract concentrations on the inhibition zone diameter of ***S. aureus***.

**Comparison**		**Mean Difference**	**Std. Error**	P	**95% Confidence Interval**	
**Conc. I (mg/ml)**	**Conc. J (mg/ml)**				**Lower Bound**	**Upper Bound**
20	10	2.32000*	.26894	.000	1.4349	3.2051
	5	6.73333*	.26894	.000	5.8482	7.6184
	2.5	9.16000*	.26894	.000	8.2749	10.0451
	1.25	11.24333*	.26894	.000	10.3582	12.1284
10	5	4.41333*	.26894	.000	3.5282	5.2984
	2.5	6.84000*	.26894	.000	5.9549	7.7251
	1.25	8.92333*	.26894	.000	8.0382	9.8084
5	2.5	2.42667*	.26894	.000	1.5416	3.3118
	1.25	4.51000*	.26894	.000	3.6249	5.3951
2.5	1.25	2.08333*	.26894	.000	1.1982	2.9684

Table S21. Tukey's HSD homogeneous subsets for the leave extract concentrations on the inhibition zone diameter of ***S. aureus***.

**Conc. (mg/mL)**	**N**	**Subset for** $\alpha = 0.05$				
		**1**	**2**	**3**	**4**	**5**
**1.25**	3	18.54	—	—	—	—
**2.5**	3	—	20.62	—	—	—
**5**	3	—	—	23.05	—	—
**10**	3	—	—	—	27.46	—
**20**	3	—	—	—	—	29.78
P		1.00	1.00	1.00	1.00	1.00

Table S22. Independent samples **t-Test** comparing flower and leave extracts at different concentrations on the inhibition zone diameter of ***E. coli***.

**Conc. (mg/mL)**	**Mean ± SD**		t	df	P	MD	$95\%\ CI$		**Levene**		**Cohen's**	**Hedges'**	**Glass's**
	**Flower**	**Leave**					**Lower**	**Upper**	F	P	D	G	Δ
**20**	21.25 ± 0.14	14.22 ± 0.30	36.88	4	<0.001	7.03	6.50	7.56	2.09	0.22	30.11	24.02	23.33
**10**	19.35 ± 0.49	12.08± 0.12	24.89	4	<0.001	7.27	6.46	8.08	5.46	0.08	20.32	16.22	62.29
**5**	15.12± 0.18	9.50± 0.28	29.70	4	<0.001	5.63	5.10	6.15	0.68	0.46	24.25	19.35	20.27
**2.5**	13.28± 0.33	0.00 ± 0.00	70.75	4	<0.001	13.28	12.76	13.80	4.13	0.11	57.77	46.09	—
**1.25**	9.01± 0.14	0.00± 0.00	111.47	4	<0.001	9.01	8.79	9.23	13.47	0.02	91.02	72.62	—

Data are expressed as mean ± standard deviation (SD). CI: confidence interval; MD: mean difference; F and P values correspond to Levene's test for equality of variances. Effect sizes were calculated using Cohen's d, Hedges' g, and Glass's Δ. A dash (—) indicates that effect size could not be computed due to zero variance in the leave extract group. All effects were evaluated at α = 0.05. Abbreviations and statistical conventions are identical across all subsequent t-test tables.

Please do not adjust margins

Table S23. Independent samples **t-Test** comparing flower and leave extracts at different concentrations on the inhibition zone diameter of ***S. aureus***.

Conc. (mg/mL)	Mean ± SD		t	df	P	MD	95% CI		Levene		Cohen's	Hedges'	Glass's
	Flower	leave					Lower	Upper	F	P	D	G	Δ
**20**	40.00 ± 0.00	29.78 ± 0.33	53.57	4	<0.001	10.22	9.69	10.75	4.797	0.094	43.74	34.90	30.93
**10**	40.00 ± 0.00	27.46 ± 0.27	81.92	4	<0.001	12.54	12.11	12.96	7.878	0.048	66.89	53.37	47.30
**5**	35.76 ± 0.49	23.05 ± 0.20	41.58	4	<0.001	12.71	11.86	13.56	3.965	0.117	33.95	27.09	62.31
**2.5**	33.21 ± 0.47	20.62 ± 0.48	32.36	4	<0.001	12.59	11.51	13.67	0.007	0.939	26.43	21.08	26.15
**1.25**	26.22 ± 0.33	18.54 ± 0.29	29.75	4	<0.001	7.69	6.97	8.40	0.055	0.827	24.29	19.38	25.68

Table S24. One-way ANOVA for evaluating the effects of flower extract concentrations and antibiotics on the inhibition zone diameter of ***E. coli***.

Source	SS	df	MS	F	P	η^2	OP
Between Groups	5172.699	20	258.635	1339.284	<0.001	0.998	1.000
Within Groups	8.111	42	0.193	—	—	—	—
Total	5180.810	62	—	—	—	—	—
Levene's Test (Median)	—	$df1 = 20$	$df2 = 12$	—	0.097	—	—

Table S25. Tukey's HSD pairwise comparisons of flower extract concentrations and antibiotics on the inhibition zone diameter of ***E. coli***.

Comparison		Mean Difference	Std. Error	P	95% Confidence Interval	
Conc. I (mg/ml)	Anti. J				Lower Bound	Upper Bound
20	CPM30	21.24667*	.35881	<0.001	19.8811	22.6123
	VA30	21.24667*	.35881	<0.001	19.8811	22.6123
	AMC20	21.24667*	.35881	<0.001	19.8811	22.6123
	COT25	-3.12000-*	.35881	<0.001	-4.4856-	-1.7544-
	GEN10	3.82000*	.35881	<0.001	2.4544	5.1856
	CN30	21.24667*	.35881	<0.001	19.8811	22.6123
	LE5	7.58000*	.35881	<0.001	6.2144	8.9456
	IPM10	-6.72000-*	.35881	<0.001	-8.0856-	-5.3544-
	AMP10	21.24667*	.35881	<0.001	19.8811	22.6123
	TOB10	10.78000*	.35881	<0.001	9.4144	12.1456
	CD2	21.24667*	.35881	<0.001	19.8811	22.6123
	DOX30	9.78000*	.35881	<0.001	8.4144	11.1456
	AK30	5.64667*	.35881	<0.001	4.2811	7.0123
	CIP5	21.24667*	.35881	<0.001	19.8811	22.6123
	AZM15	4.74667*	.35881	<0.001	3.3811	6.1123
	TE30	21.24667*	.35881	<0.001	19.8811	22.6123
10	CPM30	19.35000*	.35881	<0.001	17.9844	20.7156
	VA30	19.35000*	.35881	<0.001	17.9844	20.7156
	AMC20	19.35000*	.35881	<0.001	17.9844	20.7156
	COT25	-5.01667-*	.35881	<0.001	-6.3823-	-3.6511-
	GEN10	1.92333*	.35881	.001	.5577	3.2889
	CN30	19.35000*	.35881	<0.001	17.9844	20.7156
	LE5	5.68333*	.35881	<0.001	4.3177	7.0489
	IPM10	-8.61667-*	.35881	<0.001	-9.9823-	-7.2511-
	AMP10	19.35000*	.35881	<0.001	17.9844	20.7156
	TOB10	8.88333*	.35881	<0.001	7.5177	10.2489
	CD2	19.35000*	.35881	<0.001	17.9844	20.7156
	DOX30	7.88333*	.35881	<0.001	6.5177	9.2489
	AK30	3.75000*	.35881	<0.001	2.3844	5.1156
	CIP5	19.35000*	.35881	<0.001	17.9844	20.7156
	AZM15	2.85000*	.35881	<0.001	1.4844	4.2156
	TE30	19.35000*	.35881	<0.001	17.9844	20.7156
5	CPM30	15.12333*	.35881	<0.001	13.7577	16.4889
	VA30	15.12333*	.35881	<0.001	13.7577	16.4889
	AMC20	15.12333*	.35881	<0.001	13.7577	16.4889

This journal is © The Royal Society of Chemistry 20xx

Please do not adjust margins

	COT25	-9.24333-*	.35881	<0.001	-10.6089-	-7.8777-
	GEN10	-2.30333-*	.35881	<0.001	-3.6689-	-.9377-
	CN30	15.12333*	.35881	<0.001	13.7577	16.4889
	LE5	1.45667*	.35881	<0.001	.0911	2.8223
	IPM10	-12.84333-*	.35881	<0.001	-14.2089-	-11.4777-
	AMP10	15.12333*	.35881	<0.001	13.7577	16.4889
	TOB10	4.65667*	.35881	<0.001	3.2911	6.0223
	CD2	15.12333*	.35881	<0.001	13.7577	16.4889
	DOX30	3.65667*	.35881	<0.001	2.2911	5.0223
	AK30	-.47667-	.35881	.998	-1.8423-	.8889
	CIP5	15.12333*	.35881	<0.001	13.7577	16.4889
	AZM15	-1.37667-*	.35881	.046	-2.7423-	-.0111-
	TE30	15.12333*	.35881	<0.001	13.7577	16.4889
2.5	CPM30	13.27667*	.35881	<0.001	11.9111	14.6423
	VA30	13.27667*	.35881	<0.001	11.9111	14.6423
	AMC20	13.27667*	.35881	<0.001	11.9111	14.6423
	COT25	-11.09000-*	.35881	<0.001	-12.4556-	-9.7244-
	GEN10	-4.15000-*	.35881	<0.001	-5.5156-	-2.7844-
	CN30	13.27667*	.35881	<0.001	11.9111	14.6423
	LE5	-.39000-	.35881	1.000	-1.7556-	.9756
	IPM10	-14.69000-*	.35881	<0.001	-16.0556-	-13.3244-
	AMP10	13.27667*	.35881	<0.001	11.9111	14.6423
	TOB10	2.81000*	.35881	<0.001	1.4444	4.1756
	CD2	13.27667*	.35881	<0.001	11.9111	14.6423
	DOX30	1.81000*	.35881	.001	.4444	3.1756
	AK30	-2.32333-*	.35881	<0.001	-3.6889-	-.9577-
	CIP5	13.27667*	.35881	<0.001	11.9111	14.6423
	AZM15	-3.22333-*	.35881	<0.001	-4.5889-	-1.8577-
	TE30	13.27667*	.35881	<0.001	11.9111	14.6423
1.25	CPM30	9.01000*	.35881	<0.001	7.6444	10.3756
	VA30	9.01000*	.35881	<0.001	7.6444	10.3756
	AMC20	9.01000*	.35881	<0.001	7.6444	10.3756
	COT25	-15.35667-*	.35881	<0.001	-16.7223-	-13.9911-
	GEN10	-8.41667-*	.35881	<0.001	-9.7823-	-7.0511-
	CN30	9.01000*	.35881	<0.001	7.6444	10.3756
	LE5	-4.65667-*	.35881	<0.001	-6.0223-	-3.2911-
	IPM10	-18.95667-*	.35881	<0.001	-20.3223-	-17.5911-
	AMP10	9.01000*	.35881	<0.001	7.6444	10.3756
	TOB10	-1.45667-*	.35881	.026	-2.8223-	-.0911-
	CD2	9.01000*	.35881	<0.001	7.6444	10.3756
	DOX30	-2.45667-*	.35881	<0.001	-3.8223-	-1.0911-
	AK30	-6.59000-*	.35881	<0.001	-7.9556-	-5.2244-
	CIP5	9.01000*	.35881	<0.001	7.6444	10.3756
	AZM15	-7.49000-*	.35881	<0.001	-8.8556-	-6.1244-
	TE30	9.01000*	.35881	<0.001	7.6444	10.3756

Table S26. Tukey's HSD homogeneous subsets for the flower extract concentrations and antibiotics on the inhibition zone diameter of ***E. coli***.

Group / Treatment	N	Subset for $\alpha = 0.05$										
		1	2	3	4	5	6	7	8	9	10	11
**CPM30**	3	0.00	—	—	—	—	—	—	—	—	—	—
**VA30**	3	0.00	—	—	—	—	—	—	—	—	—	—
**AMC20**	3	0.00	—	—	—	—	—	—	—	—	—	—
**CN30**	3	0.00	—	—	—	—	—	—	—	—	—	—
**AMP10**	3	0.00	—	—	—	—	—	—	—	—	—	—
**CD2**	3	0.00	—	—	—	—	—	—	—	—	—	—
**CIP5**	3	0.00	—	—	—	—	—	—	—	—	—	—
**TE30**	3	0.00	—	—	—	—	—	—	—	—	—	—
**1.25 mg/ml**	3	—	9.01	—	—	—	—	—	—	—	—	—
**TOB10**	3	—	—	10.47	—	—	—	—	—	—	—	—
**DOX30**	3	—	—	11.47	—	—	—	—	—	—	—	—
**2.5 mg/ml**	3	—	—	—	13.28	—	—	—	—	—	—	—
**LE5**	3	—	—	—	13.67	—	—	—	—	—	—	—

Please do not adjust margins

**5 mg/ml**	3	—	—	—	—	15.12	—	—	—	—	—	—
**AK30**	3	—	—	—	—	15.60	15.60	—	—	—	—	—
**AZM15**	3	—	—	—	—	—	16.50	16.50	—	—	—	—
**GEN10**	3	—	—	—	—	—	—	17.43	—	—	—	—
**10 mg/ml**	3	—	—	—	—	—	—	—	19.35	—	—	—
**20 mg/ml**	3	—	—	—	—	—	—	—	—	21.25	—	—
**COT25**	3		—	—	—	—	—	—	—	—	24.37	—
**IPM10**	3	—	—	—	—	—	—	—	—	—	—	27.97
P		1.000	1.000	.418	1.000	.998	.606	.555	1.000	1.000	1.000	1.000

Table S27. One-way ANOVA for evaluating the effects of flower extract concentrations and antibiotics on the inhibition zone diameter of ***S. aureus***.

**Source**	SS	df	MS	F	P	η^2	OP
Between Groups	7196.487	23	312.891	308.085	<0.001	0.994	1.000
Within Groups	48.749	48	1.016	—	—	—	—
Total	7245.236	71	—	—	—	—	—
Levene's Test (Median)	—	$df1 = 23$	$df2 = 18$	—	0.204	—	—

Table S28. Tukey's HSD pairwise comparisons of flower extract concentrations and antibiotics on the inhibition zone diameter of ***S. aureus***.

**Comparison**		**Mean Difference**	**Std. Error**	P	**95% Confidence Interval**	
**Conc. I (mg/ml)**	**Anti. J**				**Lower Bound**	**Upper Bound**
20	CPM30	40.00000*	.82284	.000	36.8284	43.1716
	VA30	21.23333*	.82284	.000	18.0617	24.4049
	AMC20	28.76667*	.82284	.000	25.5951	31.9383
	COT25	25.73333*	.82284	.000	22.5617	28.9049
	GEN10	18.46667*	.82284	.000	15.2951	21.6383
	CN30	21.50000*	.82284	.000	18.3284	24.6716
	LE5	13.86667*	.82284	.000	10.6951	17.0383
	IPM10	14.86667*	.82284	.000	11.6951	18.0383
	AMP10	40.00000*	.82284	.000	36.8284	43.1716
	TOB10	22.30000*	.82284	.000	19.1284	25.4716
	CD2	17.63333*	.82284	.000	14.4617	20.8049
	DOX30	19.76667*	.82284	.000	16.5951	22.9383
	AK30	22.93333*	.82284	.000	19.7617	26.1049
	CIP5	17.50000*	.82284	.000	14.3284	20.6716
	AZM15	13.93333*	.82284	.000	10.7617	17.1049
	TE30	13.03333*	.82284	.000	9.8617	16.2049
10	CPM30	40.00000*	.82284	.000	36.8284	43.1716
	VA30	21.23333*	.82284	.000	18.0617	24.4049
	AMC20	28.76667*	.82284	.000	25.5951	31.9383
	COT25	25.73333*	.82284	.000	22.5617	28.9049
	GEN10	18.46667*	.82284	.000	15.2951	21.6383
	CN30	21.50000*	.82284	.000	18.3284	24.6716
	LE5	13.86667*	.82284	.000	10.6951	17.0383
	IPM10	14.86667*	.82284	.000	11.6951	18.0383
	AMP10	40.00000*	.82284	.000	36.8284	43.1716
	TOB10	22.30000*	.82284	.000	19.1284	25.4716
	CD2	17.63333*	.82284	.000	14.4617	20.8049
	DOX30	19.76667*	.82284	.000	16.5951	22.9383
	AK30	22.93333*	.82284	.000	19.7617	26.1049
	CIP5	17.50000*	.82284	.000	14.3284	20.6716
	AZM15	13.93333*	.82284	.000	10.7617	17.1049
	TE30	13.03333*	.82284	.000	9.8617	16.2049
5	CPM30	35.76000*	.82284	.000	32.5884	38.9316
	VA30	16.99333*	.82284	.000	13.8217	20.1649
	AMC20	24.52667*	.82284	.000	21.3551	27.6983
	COT25	21.49333*	.82284	.000	18.3217	24.6649
	GEN10	14.22667*	.82284	.000	11.0551	17.3983
	CN30	17.26000*	.82284	.000	14.0884	20.4316

This journal is © The Royal Society of Chemistry 20xx

Please do not adjust margins

	LE5	9.62667*	.82284	.000	6.4551	12.7983
	IPM10	10.62667*	.82284	.000	7.4551	13.7983
	AMP10	35.76000*	.82284	.000	32.5884	38.9316
	TOB10	18.06000*	.82284	.000	14.8884	21.2316
	CD2	13.39333*	.82284	.000	10.2217	16.5649
	DOX30	15.52667*	.82284	.000	12.3551	18.6983
	AK30	18.69333*	.82284	.000	15.5217	21.8649
	CIP5	13.26000*	.82284	.000	10.0884	16.4316
	AZM15	9.69333*	.82284	.000	6.5217	12.8649
	TE30	8.79333*	.82284	.000	5.6217	11.9649
2.5	CPM30	33.21000*	.82284	.000	30.0384	36.3816
	VA30	14.44333*	.82284	.000	11.2717	17.6149
	AMC20	21.97667*	.82284	.000	18.8051	25.1483
	COT25	18.94333*	.82284	.000	15.7717	22.1149
	GEN10	11.67667*	.82284	.000	8.5051	14.8483
	CN30	14.71000*	.82284	.000	11.5384	17.8816
	LE5	7.07667*	.82284	.000	3.9051	10.2483
	IPM10	8.07667*	.82284	.000	4.9051	11.2483
	AMP10	33.21000*	.82284	.000	30.0384	36.3816
	TOB10	15.51000*	.82284	.000	12.3384	18.6816
	CD2	10.84333*	.82284	.000	7.6717	14.0149
	DOX30	12.97667*	.82284	.000	9.8051	16.1483
	AK30	16.14333*	.82284	.000	12.9717	19.3149
	CIP5	10.71000*	.82284	.000	7.5384	13.8816
	AZM15	7.14333*	.82284	.000	3.9717	10.3149
	TE30	6.24333*	.82284	.000	3.0717	9.4149
1.25	CPM30	26.22333*	.82284	.000	23.0517	29.3949
	VA30	7.45667*	.82284	.000	4.2851	10.6283
	AMC20	14.99000*	.82284	.000	11.8184	18.1616
	COT25	11.95667*	.82284	.000	8.7851	15.1283
	GEN10	4.69000*	.82284	.000	1.5184	7.8616
	CN30	7.72333*	.82284	.000	4.5517	10.8949
	LE5	.09000	.82284	1.000	-3.0816-	3.2616
	IPM10	1.09000	.82284	.999	-2.0816-	4.2616
	AMP10	26.22333*	.82284	.000	23.0517	29.3949
	TOB10	8.52333*	.82284	.000	5.3517	11.6949
	CD2	3.85667*	.82284	.005	.6851	7.0283
	DOX30	5.99000*	.82284	.000	2.8184	9.1616
	AK30	9.15667*	.82284	.000	5.9851	12.3283
	CIP5	3.72333*	.82284	.007	.5517	6.8949
	AZM15	.15667	.82284	1.000	-3.0149-	3.3283
	TE30	-.74333-	.82284	1.000	-3.9149-	2.4283
0.625	CPM30	22.14000*	.82284	.000	18.9684	25.3116
	VA30	3.37333*	.82284	.026	.2017	6.5449
	AMC20	10.90667*	.82284	.000	7.7351	14.0783
	COT25	7.87333*	.82284	.000	4.7017	11.0449
	GEN10	.60667	.82284	1.000	-2.5649-	3.7783
	CN30	3.64000*	.82284	.010	.4684	6.8116
	LE5	-3.99333-*	.82284	.003	-7.1649-	-.8217-
	IPM10	-2.99333-	.82284	.087	-6.1649-	.1783
	AMP10	22.14000*	.82284	.000	18.9684	25.3116
	TOB10	4.44000*	.82284	.000	1.2684	7.6116
	CD2	-.22667-	.82284	1.000	-3.3983-	2.9449
	DOX30	1.90667	.82284	.788	-1.2649-	5.0783
	AK30	5.07333*	.82284	.000	1.9017	8.2449
	CIP5	-.36000-	.82284	1.000	-3.5316-	2.8116
	AZM15	-3.92667-*	.82284	.003	-7.0983-	-.7551-
	TE30	-4.82667-*	.82284	.000	-7.9983-	-1.6551-
0.312	CPM30	15.16333*	.82284	.000	11.9917	18.3349
	VA30	-3.60333-*	.82284	.011	-6.7749-	-.4317-
	AMC20	3.93000*	.82284	.003	.7584	7.1016
	COT25	.89667	.82284	1.000	-2.2749-	4.0683
	GEN10	-6.37000-*	.82284	.000	-9.5416-	-3.1984-
	CN30	-3.33667-*	.82284	.029	-6.5083-	-.1651-
	LE5	-10.97000-*	.82284	.000	-14.1416-	-7.7984-
	IPM10	-9.97000-*	.82284	.000	-13.1416-	-6.7984-

This journal is © The Royal Society of Chemistry 20xx

Please do not adjust margins

Please do not adjust margins

	AMP10	15.16333*	.82284	.000	11.9917	18.3349
	TOB10	-2.53667-	.82284	.288	-5.7083-	.6349
	CD2	-7.20333-*	.82284	.000	-10.3749-	-4.0317-
	DOX30	-5.07000-*	.82284	.000	-8.2416-	-1.8984-
	AK30	-1.90333-	.82284	.790	-5.0749-	1.2683
	CIP5	-7.33667-*	.82284	.000	-10.5083-	-4.1651-
	AZM15	-10.90333-*	.82284	.000	-14.0749-	-7.7317-
	TE30	-11.80333-*	.82284	.000	-14.9749-	-8.6317-
0.156	CPM30	12.02333*	.82284	.000	8.8517	15.1949
	VA30	-6.74333-*	.82284	.000	-9.9149-	-3.5717-
	AMC20	.79000	.82284	1.000	-2.3816-	3.9616
	COT25	-2.24333-	.82284	.512	-5.4149-	.9283
	GEN10	-9.51000-*	.82284	.000	-12.6816-	-6.3384-
	CN30	-6.47667-*	.82284	.000	-9.6483-	-3.3051-
	LE5	-14.11000-*	.82284	.000	-17.2816-	-10.9384-
	IPM10	-13.11000-*	.82284	.000	-16.2816-	-9.9384-
	AMP10	12.02333*	.82284	.000	8.8517	15.1949
	TOB10	-5.67667-*	.82284	.000	-8.8483-	-2.5051-
	CD2	-10.34333-*	.82284	.000	-13.5149-	-7.1717-
	DOX30	-8.21000-*	.82284	.000	-11.3816-	-5.0384-
	AK30	-5.04333-*	.82284	.000	-8.2149-	-1.8717-
	CIP5	-10.47667-*	.82284	.000	-13.6483-	-7.3051-
	AZM15	-14.04333-*	.82284	.000	-17.2149-	-10.8717-
	TE30	-14.94333-*	.82284	.000	-18.1149-	-11.7717-

Table S29. Tukey's HSD homogeneous subsets for the flower extract concentrations and antibiotics on the inhibition zone diameter of ***S. aureus***.

Group / Treatment	N	Subset for $\alpha = 0.05$											
		1	2	3	4	5	6	7	8	9	10	11	12
**CPM30**	3	0.00	—	—	—	—	—	—	—	—	—	—	—
**AMP10**	3	0.00	—	—	—	—	—	—	—	—	—	—	—
**AMC20**	3	—	11.23	—	—	—	—	—	—	—	—	—	—
**0.156 mg/ml**	3	—	12.02	12.02	—	—	—	—	—	—	—	—	—
**COT25**	3	—	14.27	14.27	14.27	—	—	—	—	—	—	—	—
**0.312 mg/ml**	3	—	—	15.16	15.16	15.16	—	—	—	—	—	—	—
**AK30**	3	—	—	—	17.07	17.07	17.07	—	—	—	—	—	—
**TOB10**	3	—	—	—	—	17.70	17.70	—	—	—	—	—	—
**CN30**	3	—	—	—	—	—	18.50	18.50	—	—	—	—	—
**VA30**	3	—	—	—	—	—	18.77	18.77	—	—	—	—	—
**DOX30**	3	—	—	—	—	—	20.23	20.23	20.23	—	—	—	—
**GEN10**	3	—	—	—	—	—	—	21.53	21.53	—	—	—	—
**0.625 mg/ml**	3	—	—	—	—	—	—	—	22.14	22.14	—	—	—
**CD2**	3	—	—	—	—	—	—	—	22.37	22.37	—	—	—
**CIP5**	3	—	—	—	—	—	—	—	22.50	22.50	—	—	—
**IPM10**	3	—	—	—	—	—	—	—	—	25.13	25.13	—	—
**AZM15**	3	—	—	—	—	—	—	—	—	—	26.07	—	—
**LE5**	3	—	—	—	—	—	—	—	—	—	26.13	—	—
**1.25 mg/ml**	3	—	—	—	—	—	—	—	—	—	26.22	—	—
**TE30**	3	—	—	—	—	—	—	—	—	—	26.97	—	—
**2.5 mg/ml**	3	—	—	—	—	—	—	—	—	—	—	33.21	—
**5 mg/ml**	3	—	—	—	—	—	—	—	—	—	—	35.76	—
**10 mg/ml**	3	—	—	—	—	—	—	—	—	—	—	—	40.00
**20 mg/ml**	3	—	—	—	—	—	—	—	—	—	—	—	40.00
P		1.000	.077	.055	.150	.288	.051	.077	.492	.087	.837	.280	1.000

Notes and references

1 P. L. Santos, J. P. S. C. F. Matos, L. Picot, J. R. G. S. Almeida, J. S. S. Quintans and L. J. Quintans-Junior, *Food and Chemical Toxicology*, 2019, **123**, 459–469.

This journal is © The Royal Society of Chemistry 20xx

Please do not adjust margins

Please do not adjust margins

2 B. Floris, P. Galloni, V. Conte and F. Sabuzi, *Biomolecules*, 2021, **11**, 1325.

3 J. Boonen, A. Bronselaer, J. Nielandt, L. Veryser, G. De Tre and B. De Spiegeleer, *J. Ethnopharmacol.*, 2012, **142**, 563–590.

4 M. Tan, L. Zhou, Y. Huang, Y. Wang, X. Hao and J. Wang, *Nat. Prod. Res.*, 2008, **22**, 569–575.

5 D. Misra, N. N. Ghosh, M. Mandal, V. Mandal, N. Baildya, S. Mandal and V. Mandal, *Brazilian Journal of Microbiology*, 2022, **53**, 715–726.

6 E. J. Yang, Y.-S. Kim and H. C. Chang, *J. Food Prot.*, 2011, **74**, 651–657.

7 O. O. Ferreira, S. H. M. da Silva, M. S. de Oliveira and E. H. de A. Andrade, *Molecules*, 2021, **26**, 7259.

8 J.-B. Gou, Z.-Q. Li, C.-F. Li, F.-F. Chen, S.-Y. Lv and Y.-S. Zhang, *Plant Physiology and Biochemistry*, 2016, **106**, 288–294.

9 A. Ricciardelli, A. Casillo, R. Papa, D. M. Monti, P. Imbimbo, G. Vrenna, M. Artini, L. Selan, M. M. Corsaro and M. L. Tutino, *Biofouling*, 2018, **34**, 1110–1120.

10 T. Ould Bellahcen, M. Cherki, J. A. C. Sánchez, A. Cherif and A. El Amrani, *Journal of Essential Oil Bearing Plants*, 2019, **22**, 1265–1276.

11 T. Valadbeigi and S. Rashki, *Journal of Basic Research in Medical Sciences*, 2015, **2**, 1–11.

12 A. T. Mbaveng, Q. Zhao and V. Kuete, in *Toxicological Survey of African Medicinal Plants*, Elsevier, 2014, pp. 577–609.

13 J. K. Mali, Y. B. Sutar, A. R. Pahelkar, P. M. Verma and V. N. Telvekar, *Chem. Biol. Drug Des.*, 2020, **95**, 174–181.

14 A. M. M. Youssef, D. A. M. Maaty and Y. M. Al-Saraireh, *Molecules*, 2023, **28**, 630.

15 Z. Dashtizadeh and Z. Toluei, DOI:10.21203/rs.3.rs-3328206/v1.

16 M. T. Shaaban, M. F. Ghaly and S. M. Fahmi, *J. Basic Microbiol.*, 2021, **61**, 557–568.

17 S. Arora, G. Kumar and S. Meena, *Asian J. Pharm. Clin. Res*, 2017, **10**, 64–69.

18 L. Bao, H. Sun, Y. Zhao, L. Feng, K. Wu, S. Shang, J. Xu, R. Shan, S. Duan and M. Qiu, *PLoS Pathog.*, 2023, **19**, e1011764.

19 M. T. Islam, E. S. Ali, S. J. Uddin, S. Shaw, M. A. Islam, M. I. Ahmed, M. C. Shill, U. K. Karmakar, N. S. Yarla and I. N. Khan, *Food and chemical toxicology*, 2018, **121**, 82–94.

20 C. Sivaraj, R. Abhirami, M. Deepika, V. Sowmiya, K. Saraswathi and P. Arumugam, *Journal of Drug Delivery and Therapeutics*, 2019, **9**, 68–77.

21 A. Khiralla, R. Spina, M. Varbanov, S. Philippot, P. Lemiere, S. Slezack-Deschaumes, P. André, I. Mohamed, S. M. Yagi and D. Laurain-Mattar, *Microorganisms*, 2020, **8**, 1353.

Please do not adjust margins

Please do not adjust margins

22 A. A. Hamid, M. A. Aliyu, L. Z. Abubakar, A. A. Mukadam, A. Shehu, G. Egharevba, M. J. Adisa, S. O. Ajibade, A. O. Zubair and E. O. Fagbohun, *Ife Journal of Science*, 2017, **19**, 409–416.

23 E. N. Sholkamy, P. Muthukrishnan, N. Abdel-Raouf, X. Nandhini, I. B. M. Ibraheem and A. A. Mostafa, *Saudi J. Biol. Sci.*, 2020, **27**, 3208–3220.

24 M. Ghavam, A. Afzali and M. L. Manca, *Sci. Rep.*, 2021, **11**, 8027.

25 A. Arya, S. Kumar, D. Kain, V. Vandana and K. Mikawlrawng, *Journal of Herbmed Pharmacology*, 2024, **13**, 407–419.

26 R. Ahmad, C. K. Lim, N. F. Marzuki, Y.-K. Goh, K. A. Azizan, Y. K. Goh, K. J. Goh, A. B. Ramzi and S. N. Baharum, *Molecules*, 2020, **25**, 5965.

27 L. Huang, X. Zhu, S. Zhou, Z. Cheng, K. Shi, C. Zhang and H. Shao, *Toxins (Basel).*, 2021, **13**, 495.

28 Y. W. Heng, J. J. Ban, K. S. Khoo and N. W. Sit, *Ind. Crops Prod.*, 2020, **153**, 112612.

29 I. B. Momodu, E. S. Okungbowa, B. O. Agoreyo and M. M. Maliki, *Niger. J. Biotechnol.*, 2022, **38**, 25–38.

30 S. A. Lakshmi, J. P. Bhaskar, V. Krishnan, S. Sethupathy, S. Pandipriya, W. Aruni and S. K. Pandian, *J. Biotechnol.*, 2020, **317**, 59–69.

31 B. M. Ousman, O. Belmehdi, I. Boussaoudi, B. B. Otchom and S. Younes, *International Journal of Secondary Metabolite*, 2025, **12**, 429–447.

32 J. I. Achika, R. G. Ayo, J. D. Habila and A. O. Oyewale, *Sci. Afr.*, 2020, **10**, e00552.

33 P. A. Jiko, M. Mohammad, F. T. Richi, M. A. Islam, S. Alam, M. A. Taher, C. Shao, S. Wang, P. Geng and A. Al Mamun, *J. Inflamm. Res.*, 2024, 5821–5854.

34 S. Khatua, A. Pandey and S. J. Biswas, *J Pharmacogn Phytochem*, 2016, **5**, 410–413.

35 L. P. Luhata and T. Usuki, *Bioorg. Med. Chem. Lett.*, 2021, **48**, 128248.

36 S. Khatua, A. Pandey and S. J. Biswas, *J. Pharmacogn. Phytochem.*, 2016, **5**, 410–413.

37 G. T. Siwe, R. Maharjan, A. P. Amang, C. Mezui, E. N. Zondegoumba, S. S. Akhtar, M. I. Choudhary and P. V. Tan, *J. Pharm. Pharmacogn. Res.*, 2020, **8**, 135–145.

38 L. Hernández-Vázquez, J. Palazón Barandela and A. Navarro-Ocaña, *Chapter 23 in: Rao, Venketeshwer. 2012. Phytochemicals: A Global Perspective of Their Role in Nutrition and Health. IntechOpen. ISBN: 978-953-51-4317-8. DOI: 10.5772/1387 pp: 487-502.*

39 S. Johann, C. Soldi, J. P. Lyon, M. G. Pizzolatti and M. A. Resende, *Lett. Appl. Microbiol.*, 2007, **45**, 148–153.

40 L. Salih, F. Eid, M. Elhaw and A. Hamed, *Egypt. J. Chem.*, 2021, **64**, 7157–7163.

 This journal is © The Royal Society of Chemistry 20xx

Please do not adjust margins
